# Gene Expression and Fatty Acid Profiling in *Longissimus thoracis* Muscle, Subcutaneous Fat, and Liver of Light Lambs in Response to Concentrate or Alfalfa Grazing

**DOI:** 10.3389/fgene.2019.01070

**Published:** 2019-10-31

**Authors:** Elda Dervishi, Laura González-Calvo, Mireia Blanco, Margalida Joy, Pilar Sarto, R. Martin-Hernandez, Jose M. Ordovás, Magdalena Serrano, Jorge H. Calvo

**Affiliations:** ^1^Livestock Gentec, University of Alberta, Edmonton, AB, Canada; ^2^Unidad de Producción y Sanidad Animal, Centro de Investigación y Tecnología Agroalimentaria de Aragón (CITA)-Instituto Agroalimentario de Aragón (IA2) (CITA-Universidad de Zaragoza), Zaragoza, Spain; ^3^Precision Nutrition and Obesity, IMDEA-Alimentación, Madrid, Spain; ^4^Jean Mayer-USDA Human Nutrition Research Center on Aging, Tufts University, Boston, MA, United States; ^5^Departamento de Mejora Genética Animal, INIA, Madrid, Spain; ^6^ARAID, Zaragoza, Spain

**Keywords:** concentrate, alfalfa, microarray, ovine, muscle, subcutaneous fat, liver, meat quality

## Abstract

A better understanding of gene expression and metabolic pathways in response to a feeding system is critical for identifying key physiological processes and genes associated with polyunsaturated fatty acid (PUFA) content in lamb meat. The main objective of this study was to investigate transcriptional changes in *L. thoracis* (LT) muscle, liver, and subcutaneous fat (SF) of lambs that grazed alfalfa (ALF) and concentrate-fed (CON) slaughtered at 23 kg and using the Affymetrix Ovine Gene 1.1 ST whole-genome array. The study also evaluated the relationship between meat traits in LT muscle, including color, pigments and lipid oxidation during 7 days of display, α-tocopherol content, intramuscular fat (IMF) content and the fatty acid (FA) profile. Lambs that grazed on alfalfa had a greater α-tocopherol concentration in plasma than CON lambs (P < 0.05). The treatment did not affect the IMF content, meat color or pigments (P > 0.05). Grazing increased the α-tocopherol content (P < 0.001) and decreased lipid oxidation on day 7 of display (P < 0.05) in LT muscle. The ALF group contained a greater amount of conjugated linoleic acid (CLA), C18:3 n−3, C20:5 n−3, C22:5 n−3, and C22:6 n−3 than did the CON group (P < 0.05). We identified 41, 96 and four genes differentially expressed in LT muscle, liver, and subcutaneous fat, respectively. The most enriched biological processes in LT muscle were skeletal muscle tissue development, being the genes related to catabolic and lipid processes downregulated, except for *CPT1B*, which was upregulated in the ALF lambs. Animals grazing alfalfa had lower expression of desaturase enzymes in the liver (*FADS1* and *FADS2*), which regulate unsaturation of fatty acids and are directly involved in the metabolism of n−3 PUFA series. The results found in the current study showed that ingesting diets richer in n−3 PUFA might have negative effects on the *de novo* synthesis of n−3 PUFA by downregulating the *FADS1* and *FADS2* expression. However, feeding diets poorer in n−3 PUFA can promote fatty acid desaturation, which makes these two genes attractive candidates for altering the content of PUFAs in meat.

## Introduction

Public health policies recommend an increase in the intake of the n−3 polyunsaturated fatty acid (PUFA) series due to the positive impact these molecules have on human health. In addition, a decrease in the consumption of trans-fatty acids and saturated fatty acids (SFAs) is recommended because they have been associated with increased cholesterol levels (Takeuchi and Sugano, 2017; Zhu et al., 2019). Other fatty acids, such as conjugated linoleic acids (CLAs), have also received increasing attention because of their possible beneficial effects on human health (Lehnen et al., 2015; Lee et al., 2018).

Currently, increasing feed efficiency and producing lean meat without reducing the nutritional value of the meat are major challenges of the meat industry. The nutritional value of meat can be influenced by dietary and genetic effects (Scollan et al., 2014). Grass feeding improves eicosapentaenoic acid (20:5n−3, EPA), docosapentaenoic acid (DPA, 22:5n−3), and docosahexaenoic acid (22:6n−3, DHA) contents in muscle (Fisher et al., 2000; Dervishi et al., 2010; Dervishi et al., 2011) as forage increases the content of alpha-linolenic acid (18:3n−3), the precursor for DHA and EPA production (Kitessa et al., 2010). Diet has been shown to have a major impact on the intramuscular FA profile of the muscle of light lambs (Dervishi et al., 2010; González-Calvo et al., 2015a); grazing increases the PUFA content of the n−3 series and conjugated fatty acids (CLAs) when compared to that with concentrate feeding. In *semitendinosus* muscle, genes related to adipogenesis are upregulated in concentrate-fed lambs, whereas *CPT1B* gene expression, related to the β-oxidation process, is upregulated in grazing lambs (Dervishi et al., 2011). However, the expression of genes implicated in lipid metabolism is not similar in the *longissimus* muscle of grazing and concentrate-fed lambs (González-Calvo et al., 2015a). These results demonstrate that the diet/feeding system has a differential effect on gene expression in different animal tissues. It has also been demonstrated that fiber type composition in skeletal muscle (the relative amounts of fast versus slow twitch fibers) affected the gene expression profiles among different muscle under the same environment (Terry et al., 2018). Therefore, a better understanding of the genes and metabolic pathways in response to the feeding system is critical for identifying key physiological processes and genes associated with lipid metabolism, especially for the n−3 PUFA series. A deeper understanding of the gene regulation of n−3 levels in lamb meat may help in designing new strategies for the production of healthier meat and satisfying consumers’ demand.

The combination of technologies such as fatty acids and gene profiling provides a powerful tool for discovering gene expression changes associated with meat quality traits and for discovering genes contributing to fatty acid content variation in meat. The main objective of this study was to investigate the fatty acid profile and transcriptional changes in the LT muscle, liver, and subcutaneous fat (SF) of lambs grazing on alfalfa pasture and receiving concentrate using the Affymetrix Ovine Gene 1.1 ST whole-genome array. Furthermore, we aimed to identify novel genes that may play important roles in the metabolism of PUFAs that may be associated with meat quality traits.

## Material and Methods

### Ethics Statement

All experimental procedures, including the care of animals and euthanasia, were performed in accordance with the guidelines of the European Union and Spanish regulations for the use and care of animals in research and were approved by the Animal Welfare Committee of the Centro de Investigación y Tecnología Agroalimentaria (CITA) (protocol number 2009-01_MJT). In all cases, euthanasia was performed by penetrating captive bolt followed by immediate exsanguination.

### Animals and Sample Collection

Fourteen pairs of ewe-single reared male lambs of the Rasa Aragonesa breed grazed continuously during lactation in alfalfa pastures. The lambs had *ad libitum* access to a concentrate during lactation. Seven pairs of ewe-lambs were not weaned but remained grazing alfalfa with their mothers from birth until the slaughter of the lambs (23 ± 0.4 kg) (ALF group). The other seven lambs were weaned (48 ± 0.9 days of age) and then fed a basal concentrate for 24 (± 2.6) days until slaughter at 23 kg (CON group). These lambs were the same as those described in González-Calvo et al. (2017), and were reared alongside the ALF group. Lambs belonging to ALF treatment received dams’ milk, fresh alfalfa (grazing) and commercial concentrate, the same that was offered to CON treatment during the experimental period. The average concentrate intake of the CON and ALF groups during the experimental period was 24.3 and 7.4 kg per lamb, respectively. The weaning weight of CON treatment animals was 11.6 ± 1.91 kg BW and the weight of the alfalfa lambs at the same moment of the weaning of CON treatment was 12.8 ± 1.35 kg BW. The ingredients, chemical composition and FA composition of the feedstuffs are shown in **Table 1**. The experimental procedures, composition of diets, management of the animals and sample details for each group are described in detail in Ripoll et al. (2013). Blood samples were obtained weekly in test tubes containing heparin from the jugular vein. Samples were centrifuged at 3,500 rpm for 20 min, and plasma was stored at −80°C until α-tocopherol and triacylglycerols (TG), cholesterol, low density lipoprotein-cholesterol (LDL-cholesterol) and high density lipoprotein-cholesterol (HDL-cholesterol) analyses.

**Table 1:** Ingredients and chemical composition of the feedstuffs used in the experiment.

Item	Treatment^1^	
	ALF	CON
**Ingredients (%)**		
Barley		40.87
Corn		14.95
Wheat		20.08
Soyabean meal		19.78
Salt		0.39
Carbonate		1.64
Mineral–vitamin mixture		1.20
Fat		1
**Chemical composition** **^2^**		
g DM/kg as fed	24.6	88.6
CP, g/kg DM	154	175
CF, g/kg DM		38
NDF, g/kg DM	326	180
ADF, g/kg DM	204	45
α-tocopherol, mg/kg DM DM	154	27**^3^**
**FA composition (g/100 g FA)** **^4^**		
C16:0	17.7	24.35
C18:0	3.02	2.91
C18:1 c9	4.52	25.63
C18:2 n−6	22.28	38.86
C18:3 n−3	37.93	3.14
C20:0	4.46	0.33
C22:0	3.03	0

^1^CON: commercial concentrate; ALF: unweaned lambs grazing alfalfa plus commercial concentrate.

^2^DM, dry matter; CP, crude protein; CF, crude fat; NDF, neutral detergent fibre; ADF, acid detergent fibre.

^3^As mg dl-α-tocopheryl acetate/kg DM.

^4^Fatty acid composition expressed as the percentage of total fatty acid methyl esters.

All the lambs were slaughtered when they reached 22–24 kg of slaughter weight (SW) according to the specifications of Ternasco de Aragón Protected Geographical Indication (Regulation (EC) No. 1107/96) that stipulates that lambs must be younger than 90 days old with a SW between 22 and 24 kg. The lambs were slaughtered using EU laws in the same commercial abattoir, and the carcasses were hung by the Achilles tendon and chilled for 24 h at 4°C in total darkness. The slaughter age, slaughter weight, and growth rate of the two management strategies are presented in **Supplementary Table 1**.

Just after slaughter, a sample of the LT muscle from the 12th thoracic *vertebra*, a sample of SF between the atlas and axis *cervical vertebrae* and a sample of the liver were excised, frozen in liquid nitrogen and stored at −80°C until RNA isolation.

### Chemical Analyses

#### Intramuscular Fat (IMF)

The intramuscular fat content was quantified using the Ankom procedure (AOAC, 2000) with an Ankom extractor (model XT10, Ankom Technology, New York, USA).

#### Fatty Acid Determination

Both muscle and feed fatty acids were determined as described in González-Calvo et al. (2015a). Feed samples were Soxhlet extracted (Sukhija and Palmquist, 1988), and muscle samples were determined according to Bligh and Dyer (1959) with the modifications described in González-Calvo et al. (2015a). The individual FA contents were expressed as weight percentages (g/100 g of FAME). The total amount of SFA, monounsaturated FA (MUFA), PUFA, n−6 PUFA and n−3 PUFA contents and their associated ratios (PUFA : SFA and n−6:n−3) were determined.

#### Analysis of α-Tocopherol, TG, LDL-Cholesterol, HDL-Cholesterol, and Cholesterol in Plasma

Alpha-tocopherol in plasma was determined by liquid extraction in duplicate as described in González-Calvo et al. (2015b). Triacylglycerols, cholesterol, LDL-cholesterol and HDL-cholesterol were determined using an automatic analyzer (Gernonstar, RAL, Barcelona, España). The reagent manufacturer was RAL (Técnica para el Laboratorio, S.A. Sant Joan Despí, Barcelona, Spain). The mean intra-assay coefficients of variation were 0.99–1.57%, 0.76–1.22%, 0.63–0.67%, and 0.8–1.06% for TG, cholesterol, LDL-cholesterol and HDL-cholesterol, respectively. The interassay coefficients of variation were 3.15–7.77%, 4.36–6.91%, 1.29–1.45% and 2.71–4.60% for the same metabolites.

#### Analysis of **α**-Tocopherol Concentration, TBARS and Metmyoglobin Formation in Muscle

After it was chilled, a piece of the LT muscle between the 4th and the 6th lumbar *vertebrae* was vacuum-packed and kept at −20°C in darkness until the α-tocopherol analysis. The α-tocopherol concentration was determined by liquid extraction as described in González-Calvo et al. (2015b). A portion of the loin between the 7th and the 13th thoracic *vertebrae* was used to measure the color (metmyoglobin content, MMb) and lipid oxidation analysis (thiobarbituric acid-reactive substance, TBARS), and were quantified at 7 days after being maintained in darkness at 4°C. The LT muscle color and LT intramuscular fat TBARS analysis were measured as described in González-Calvo et al. (2015b). Briefly, the relative content of metmyoglobin (MMb) was estimated by the K/S_572/525_ ratio (Hunt, 1980). This ratio decreases when the MMb content increases. The TBARS analysis was performed using the procedure reported by Pfalzgraf et al. (1995). The TBARS values are expressed as milligrams of malonaldehyde (MDA) kg^−1^ of muscle.

### RNA Isolation and Assessment of RNA Integrity

Total RNA was extracted from approximately 500 mg of LT muscle, SF, and liver using RNeasy Tissue mini kits (QIAGEN, Madrid, Spain) following the manufacturer’s protocol. Prior to microarray analysis, RNA integrity and quality were assessed by an RNA 6000 Nano LabChip on an Agilent 2100 Bioanalyzer and quantified using a nanophotometric spectrophotometer (Implen, Madrid, Spain). All RNA integrity number (RIN) values were above 8.

### Microarray Hybridization and Data Processing

RNA samples (n = 14, seven samples from each treatment) were analyzed using the Ovine Gene 1.1 ST Array Strip (Affymetrix, High Wycombe, UK). Microarray hybridization and scanning were performed at the Functional Genomics Core Facility (Institute for Research in Biomedicine, IRB Barcelona, Spain) following the recommendations of the manufacturer. Scanned images (DAT files) were transformed into intensities (CEL files) by Affymetrix GeneChip Operating Software (GCOS). The overall array intensity was normalized between arrays to correct for systematic bias in the data and to remove the impact of nonbiological influences on biological data. The imported data were analyzed at the gene level, with exons summarized to genes, using the mean expression of all the exons of a gene. Normalization was carried out with the Robust Multi-Array Average (RMA) algorithm using quantile normalization, median polish probe summarization, and log2 probe transformation. The datasets supporting the results and discussed in this publication have been deposited in the NCBI Gene Expression Omnibus repository (Barrett et al., 2012) and are accessible through GEO Series accession numbers GSE63774 (LT muscle and SF) and GSE125661 (liver). The datasets for LT muscle and SF in CON group were previously presented in González-Calvo et al. (2017).

### Validation of Microarray Data by Real-Time Quantitative PCR Analysis (RT-qPCR)

One microgram of RNA from each sample was treated with DNAse (Invitrogen, Carlsbad, CA, USA), and single-stranded cDNA was synthesized using the SuperScript^®^III Reverse Transcriptase kit (Invitrogen, Carlsbad, CA, USA), following the manufacturer’s recommendations. Specific exon-spanning primers for genes were generated and confirmed for specificity using BLAST (National Center for Biotechnology Information: http://www.ncbi.nlm.nih.gov/BLAST/). Before performing the real-time PCRs, a conventional PCR was performed for all genes to test the primers and to verify the amplified products. The PCR products were sequenced to confirm gene identity using an ABI Prism 3700 (Applied Biosystems, Madrid, Spain) with standard protocols. Homology searches were performed with BLAST to verify the identity of the amplified fragments. The real-time PCR was carried out in a 10 μl PCR total reaction mixture containing SYBR Green Master Mix: SYBR Premix Ex Taq II (Tli RNase H Plus, Takara, Sumalsa, Zaragoza, Spain). Reactions were run in triplicate on an ABI Prism 7500 platform (Applied Biosystem, Madrid, Spain) following the manufacturer’s cycling parameters. Standard curves for each gene were generated to calculate the amplification efficiency through a 4-fold serial dilution of cDNA pooled from LT muscle, liver and SF. The efficiency (E) of PCR amplification for each gene was calculated using the standard curve method (E = 10^(−1/slope)^). Two “connector samples” were replicated in all plates to remove technical variation from this source of variability. The annealing temperatures, primer concentrations, and primer sequences for GOIs (Genes of interest: *CPT1B*, *MYOD1*, *MSTN*, *ABCC4*, *IGF1R* and *PLA2G16* for LT muscle; *METTL1* for SF; and *FADS1*, *FADS2*, *ACACA*, *SCD*, *SQLE*, *IER3*, *SLC19A1* and *THRSP* for liver tissue) and reference genes (*GUSB* and *YWHAZ* for LT muscle and SF; and *RPL37*, *GUSB* and *RPL19* for liver) are described in **Supplementary Table 2**. These reference genes for LT muscle and SF were chosen because they were the most stable in these tissues in previous studies (González-Calvo et al., 2014). Five candidate reference genes (*B2M*, *YWHAZ*, *RPL37*, *RPL19*, and *GUSB*) were tested for liver tissue. Determinations of the gene expression stability of liver genes included in this study were calculated using NormFinder to select the best reference genes (Andersen et al., 2004).

### Statistical Analysis

#### Statistical Analysis of the Performance, Concentrations of TG, LDL-Cholesterol, HDL-Cholesterol, and Cholesterol in Plasma, and Meat Quality Characteristics in LT Muscle

Statistical analysis of the performance, the plasma metabolites and lipid oxidation of LT muscle (TBARS) was performed using the SAS statistical package v. 9.3 (SAS Institute, Cary NC, USA). The concentration of analytes in plasma, lipid oxidation levels and meat color and pigments were analyzed using mixed models for repeated measurements based on Kenward-Roger’s adjusted degrees of freedom solution for repeated measures including the management strategy (CON and ALF), the week/time of display and its interaction as fixed effects and the lamb as the random effect. A first-order autoregressive structure with heterogeneous variances for each date was used to model heterogeneous residual error.

The weight gain, age, weight at slaughter and IMF in LT muscle were analyzed using a general lineal model (GLM) with the treatment as a fixed factor. The content of α-tocopherol and the fatty acid profile of LT muscle were analyzed with a GLM with the treatment as a fixed factor and the slaughter age (SA) as a covariate. The results were expressed as least square means (LSM) ± the standard error (SE) values, and the differences were tested at a level of significance of 0.05 with the t statistic. The Tukey *post hoc* test was used to evaluate differences between treatments.

#### Microarray Gene Expression Statistical Analysis

##### Identification of Differentially Expressed Genes by Microarray Analysis in LT Muscle and SF

Normalized data were further analyzed using Babelomics (http://babelomics.bioinfo.cipf.es/graph.html) and MetaboAnalyst software (Xia et al., 2009). Genes showing a statistically significant value of the Limma test (*P* < 0.01) were screened out as differentially expressed between treatments. Significant genes were annotated based on similarity scores in blastn comparisons of Affymetrix transcript cluster sequences against ovine sequences in GenBank. A second method, significance analysis of microarray (SAM), was used to identify and reconfirm differentially expressed genes in ALF–CON comparisons. Details of the protocol are described in González-Calvo et al. (2017).

##### Multivariate Analysis of Gene Expression and Hierarchical Clustering Analysis (HCA)

Multivariate and cluster analysis was performed using MetaboAnalyst according to Xia et al. (2009). Principal components analysis (PCA) was used to cluster the samples based on the selected gene expression profile for each tissue. Hierarchical clustering analysis for gene expression was performed using all genes and only the significant genes for each tissue. Details are described in González-Calvo et al. (2017).

#### Statistical Analysis of Gene Expression Validated by RT-qPCR

The corresponding mRNA levels were measured and analyzed by their quantification cycle (Cq). The statistical methodology to analyze differences in the expression rate was carried out following the method proposed by Steibel et al. (2009). The mixed model fitted was as follows:

$$y_{\mathrm{rigkm}}=TG_{\mathrm{gi}}+P_{k}+b_{1}{\left(\mathrm{IMF}\right)}_{m}+b_{2}{\left(\mathrm{SA}\right)}_{m}+A_{m}+e_{\mathrm{rigkm}}$$

where *y* *_rigkm_* is the *C*_q_ value (transformed data taking into account E < 2) obtained from the thermocycler software for the gth gene (GOIs and reference genes) from the rth well (reactions were run in triplicate) in the kth plate corresponding to the mth animal and to the ith treatment (CON and ALF); TG_gi_ is the fixed interaction among the ith treatment and the gth gene (T is the effect of the ith treatment, and G is the effect of the gth gene); P_k_ is the fixed effect of the kth plate; IMF_m_ and SA_m_ are the effects of intramuscular fat (only used in LT muscle tissue gene expression) and the slaughter age of the mth animal, respectively, included as covariates; A_m_ is the random effect of the mth animal from where samples were collected (A_m_∼(0,σ^2^_A_)); and e_rigkm_ is the random residual. Gene-specific residual variance (heterogeneous residual) was fitted to the gene by treatment effect (*e* *_rigkm_*∼N(0, σ^2^_egi_).

To test differences (*diff* *_GOI_*) in the expression rate of the target genes between treatments and to obtain fold change (FC) values from the estimated TG differences, the approach suggested in Steibel et al. (2009) was used. The significance of the *diff* *_GOI_* estimates was determined with the t statistic. Additionally, asymmetric 95% confidence intervals (upper and lower) were calculated for each FC value using the standard error (SE) of *diff* *_GOI_*.

### Functional Annotation Analyses

The Database for Annotation, Visualization and Integrated Discovery (DAVID) v6.7b (Huang et al., 2008) was used to determine pathways and processes of major biological significance and importance through the Functional Annotation Cluster (FAC) tool based on the Gene Ontology (GO) annotation function. DAVID FAC analysis was performed with the gene lists obtained after SAM analysis. Medium stringency EASE score parameters were selected to indicate confident enrichment scores of functional significance and importance of the given pathways and processes investigated. An enrichment score of 1.3 was employed as the threshold for cluster significance. The ClueGO plug-in (Bindea et al. 2009) and Cytoscape program (Shannon et al., 2003) were used to group genes according to the similarity of the biological processes in which they are involved. The relationships between the n−3 PUFA series in muscle and gene expression in liver were visualized using the Metscape plugin (Karnovsky et al., 2012) in Cytoscape (Shanon et al., 2003). Thirty significant genes (out of 96) and nine (out of 13) significant compounds were mapped to KEGG IDs. The file containing the list of genes and metabolites, their fold change and P-values was loaded in Metscape to generate a compound-gene network.

## Results

### Lambs Performance and α-Tocopherol, TG, LDL-Cholesterol, HDL-Cholesterol and Cholesterol Concentrations in Plasma

No differences were found in the slaughter weight and age (SA) or average daily gain (ADG) from birth to slaughter between treatments (**Supplementary Table 1**). Type III tests of the fixed effects (treatment and day) on blood parameters are shown in **Supplementary Table 3**.

The concentrations of α-tocopherol, cholesterol, LDL-cholesterol and HDL-cholesterol in plasma were affected by the interaction between the treatment and the day (P < 0.05 to < 0.01). Meanwhile, the concentration of TG in plasma was affected only by the treatment (P < 0.01).

**Figure 1:**
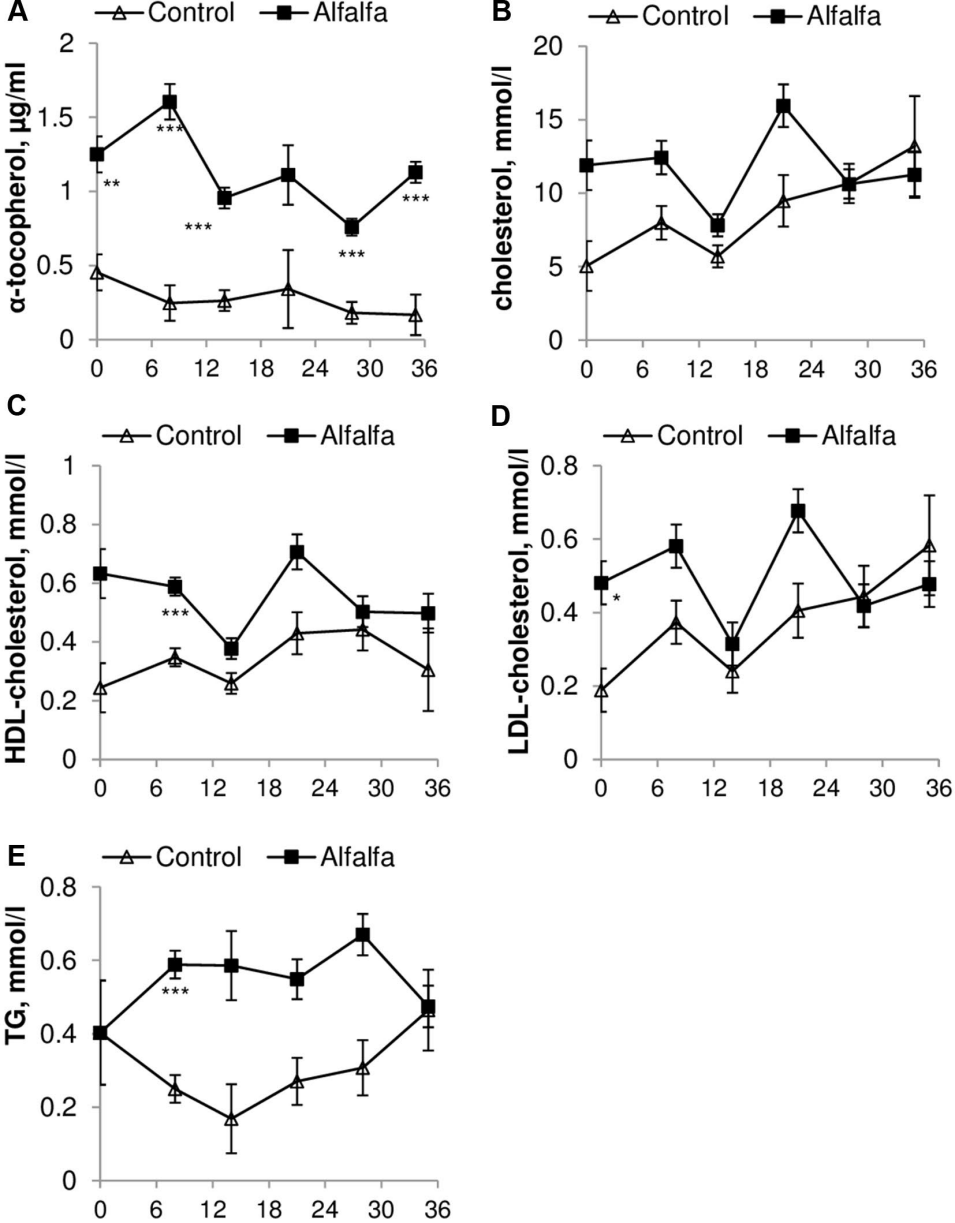
Concentration of **(A)** α-tocopherol, **(B)** cholesterol, **(C)** HDL-cholesterol, **(D)** LDL-cholesterol and **(E)** TG during the entire experimental period compared with those in the CON group.

Grazing animals (ALF group) had a greater concentration of α-tocopherol (P < 0.0001) and similar cholesterol (P = 0.056) contents throughout the experimental period when compared to those in the CON group (**Figures 1A, B**). The HDL-cholesterol content was similar between treatments except on day 8 after weaning, and the ALF group had a greater content when compared to that in the CON group (0.58 ± 0.03 mmol/l vs. 0.34 ± 0.03 mmol/l; P < 0.05). Similarly, ALF lambs presented a greater concentration of TG on days 8 and 28 postweaning (0.58 ± 0.03 vs. 0.67 ± 0.05 and 0.25 ± 0.03 vs. 0.3073 ± 0.07 mmol/l; P < 0.05). The LDL-cholesterol content was greater in the ALF group only on day 0 (equivalent to the day of weaning in the CON group) when compared to that in the CON group (0.48 ± 0.05 vs. 0.18 ± 0.05 mmol/l; P < 0.05) (**Figure 1**).

### Meat Characteristics in *Longissimus thoracis* Muscle

Intramuscular fat content, meat color and pigments were not different between treatments (**Supplementary Table 4**; *P* > 0.05); however, ALF lambs had a greater content of α-tocopherol in LT muscle when compared with that in the CON group (2.38 ± 0.17 vs. 0.48 ± 0.17; *P* < 0.05) (**Supplementary Table 3**). Lipid oxidation was affected by the interaction between the treatment and the day of display (P < 0.01). Lipid oxidation was similar in the first days of display (P > 0.05), but ALF lambs had lower oxidation than did CON lambs on day 7 of display (P < 0.05).

Regarding the fatty acid profile, LT muscle from the ALF group had a greater content of capric (C10:0) and arachidic (C20:0) acids (**Table 2**; P < 0.05) and tended to have a greater content of margaric acid (C17:0) and stearic acid (C18:0) (0.1 < P > 0.05). However, the total SFA content was not different between treatments (P > 0.05; **Table 2**).

**Table 2:** Effect of the treatment on the content of α-tocopherol and fatty acid (FA) composition of LT muscle in Rasa Aragonesa lambs.

Compounds^1^	Treatment^2^		P-value	
	CON	ALF	Feeding	Slaughter Age
α-tocopherol, mg/kg muscle	0.48 ± 0.17	2.38 ± 0.17	0.0001	0.38
C8:0	0.02 ± 0.008	0.04 ± 0.008	0.15	0.07
C10:0	0.17 ± 0.016	0.25 ± 0.016	0.0047	0.13
C12:0	0.42 ± 0.051	0.53 ± 0.051	0.16	0.43
C14:0	4.47 ± 0.318	5.17 ± 0.318	0.16	0.78
C16:0	24.97 ± 0.715	24.1 ± 0.715	0.42	0.41
C16:1	2.06 ± 0.127	1.83 ± 0.127	0.24	0.32
C17:0	0.96 ± 0.069	1.15 ± 0.069	*0.08*	0.48
C17:1	0.81 ± 0.047	0.82 ± 0.047	0.90	0.35
C18:0	11.76 ± 0.364	12.87 ± 0.364	*0.06*	0.01
C18:1 n−9	41.48 ± 0.947	36.65 ± 0.947	0.005	0.29
C18:1 n−7	1.01 ± 0.135	1.21 ± 0.135	0.33	0.47
C18:2 n−6tt	0.14 ± 0.047	0.53 ± 0.047	0.0001	0.24
C18:2 n−6ct	6.6 ± 0.517	6.65 ± 0.517	0.95	0.58
C18:3 n−6	0.07 ± 0.005	0.07 ± 0.005	0.90	0.27
C18:3 n−3	0.37 ± 0.24	2.24 ± 0.24	0.0002	0.93
C20:0	0.08 ± 0.01	0.13 ± 0.01	0.004	0.34
CLA	0.59 ± 0.065	0.85 ± 0.065	0.02	0.62
C20:1 n−9	0.11 ± 0.008	0.09 ± 0.008	0.21	0.08
C22:0	0.16 ± 0.021	0.19 ± 0.021	0.36	0.92
C20:4 n−6	2.67 ± 0.37	2.01 ± 0.37	0.24	0.47
C20:3 n−3	0.001 ± 0.009	0.02 ± 0.009	0.24	0.76
C20:5 n−3	0.13 ± 0.12	0.9 ± 0.12	0.001	0.92
C24:0	0.24 ± 0.056	0.15 ± 0.056	0.31	0.78
C22:5 n−3	0.51 ± 0.114	1.05 ± 0.114	0.008	0.80
C22:6 n−3	0.21 ± 0.072	0.51 ± 0.072	0.02	0.56
Saturated FA (SFA)	43.25 ± 0.924	44.58 ± 0.924	0.35	0.10
Monounsaturated FA	45.46 ± 0.939	40.6 ± 0.939	0.005	0.25
Polyunsaturated FA (PUFA)	11.29 ± 1.187	14.82 ± 1.187	0.07	0.67
PUFA n−6	9.48 ± 0.855	9.25 ± 0.855	0.86	0.57
PUFA n−3	1.22 ± 0.521	4.72 ± 0.521	0.0008	0.93
n−6:n−3	7.67 ± 0.697	2.84 ± 0.697	0.0006	0.16
PUFA : SFA	0.26 ± 0.032	0.34 ± 0.032	0.14	0.54

^1^Fatty acid composition it is expressed as weight percentages total fatty acid methyl esters (g/100 g of FAME).

^2^CON, weaned lambs fed commercial concentrates; ALF, unweaned grazing alfalfa lambs.

The treatment did not affect palmitoleic acid (C16:1; P > 0.05), vaccenic acid (C18:1 n−7; P > 0.05) and eicosenoic acid (C20:1 n−9; P > 0.05) content but did affect the content of oleic acid (C18:1 n−9; P < 0.05) and total MUFAs, which was greater in CON than in ALF lambs (P < 0.05; **Table 2**). Regarding individual PUFA contents, the linoleic acid (C18:2 n−6), linolenic acid (C18:3 n−3), EPA (C20:5 n−3), docosapentaenoic acid (C22:5 n−3), and DHA (C22:6 n−3) contents were greater in the ALF group than in the CON group (P < 0.05). In addition, the LT muscle of ALF lambs had a greater n−3 PUFA content and a lower n−6:n−3 ratio (P < 0.001) when compared with those in CON lambs but did not affect the total PUFA content (P = 0.07).

### Microarray Gene Expression Results

#### Identification and Classification of Differentially Expressed Genes in LT Muscle, Liver, and SF

Forty-one, four and 96 genes were differentially expressed in LT muscle, SF, and liver, respectively, after SAM analysis (**Supplementary Figure 1**). In LT muscle, 41 genes were differentially expressed with an FDR = 0.002 (**Table 3**), of which 32 were downregulated and nine genes were upregulated. In the liver, 96 genes were differentially expressed (**Supplementary Table 5**), among which four genes were upregulated and 92 genes were downregulated with ALF treatment (FDR = 0.002). The top 20 significant genes in the liver are shown in **Table 4**.

**Table 3:** Differentially expressed genes in *Longissimus thoracis* muscle and fold-change in ALF–CON^1^ contrast.

Gene symbol	Gene name	q value^2^	FC^3^
*ABCC4*	ATP-binding cassette, sub-family C (CFTR/MRP), member 4	0.001	−1.42
*MYOD1*	Myogenic differentiation 1	0.001	−2.77
*NEK7*	NIMA (never in mitosis gene a)-related kinase 7	0.001	−1.28
*CDC5L*	CDC5 cell division cycle 5-like	0.001	1.15
*ANGEL1*	Angel homolog 1	0.001	−1.18
*BOLA3*	Similar to bolA-like 3; bolA homolog 3	0.001	1.20
*C8orf4*	Chromosome 8 open reading frame 4	0.001	3.30
*CHP1*	Chromo domain-containing protein 1	0.001	1.26
*CNBP*	CCHC-type zinc finger, nucleic acid binding protein	0.001	−1.22
*CPT1B*	Choline kinase beta; carnitine palmitoyltransferase 1B (muscle)	0.001	1.47
*DNAJB11*	DnaJ (Hsp40) homolog, subfamily B, member 11	0.001	1.34
*FZD7*	Frizzled homolog 7	0.001	−1.33
*HSF2*	Heat shock transcription factor 2	0.001	1.47
*IGF1R*	Insulin-like growth factor 1 receptor	0.001	−1.19
*LRTOMT*	Leucine rich transmembrane and 0-methyltransferase domain containing	0.001	−1.23
*MACROD1*	MACRO domain containing 1	0.001	−1.17
*MYLK2*	Myosin light chain kinase 2	0.001	−1.39
*NMT1*	N-myristoyltransferase 1	0.001	1.11
*PLA2G16*	Phospholipase A2, group XVI	0.001	−1.36
*PLEKHH3*	Pleckstrin homology domain containing, family H member 3	0.001	−1.60
*PRDM1*	PR domain containing 1, with ZNF domain	0.001	−1.52
*R3HCC1*	R3H domain and coiled-coil containing 1	0.001	−1.18
*RSC1A1*	Regulatory solute carrier protein, family 1, member 1	0.001	−1.25
*SPSB1*	splA/ryanodine receptor domain and SOCS box containing 1	0.001	−1.39
*TP53INP2*	Tumor protein p53 inducible nuclear protein 2	0.001	1.66
*ANK3*	Ankyrin 3, node of Ranvier (ankyrin G)	0.001	−1.25
*BCL9L*	B-cell CLL/lymphoma 9-like	0.001	−1.33
*DNPEP*	Aspartyl aminopeptidase	0.001	−1.24
*TCEA3*	Transcription elongation factor A (SII), 3	0.001	−1.27
*SLC7A8*	Solute carrier family 7, member 8	0.001	−1.98
*YPEL2*	Yippee-like 2 (Drosophila)	0.001	−1.31
*SLC8A3*	Solute carrier family 8 (sodium/calcium) exchanger), member 3	0.001	−1.91
*PLCD4*	Phospholipase C, delta 4	0.001	−1.38
*CMBL*	Carboxymethylenebutenolidase homolog	0.001	−1.44
*MYOZ1*	Myozenin 1	0.001	−1.13
*MLF2*	Myeloid leukemia factor 2	0.001	−1.17
*SF3A1*	Splicing factor 3a, subunit 1, 120 kDa	0.001	−1.17
*ALDH2*	Aldehyde dehydrogenase 2 family (mitochondrial)	0.001	−1.21
*CYP27A1*	Cytochrome P450, family 27, subfamily A, polypeptide 1	0.002	−1.26
*FBXO9*	F-box protein 9	0.002	−1.18
*MSTN*	Myostatin	0.002	−2.43

^1^CON, weaned lambs fed commercial concentrates; ALF, unweaned grazing alfalfa lambs.

^2^q value: significance level^.^

^3^FC, fold-change.

**Table 4:** Top 20 differentially expressed genes in the liver and fold-change in ALF–CON^1^ contrast.

Gene symbol	Gene name	q value^2^	FC^3^
*ACACA*	acetyl-CoA carboxylase alpha	0.001	−1.99
*ACSS2*	acyl-CoA synthetase short-chain family member 2	0.0007	−2.51
*CSAD*	cysteine sulfinic acid decarboxylase	0.0007	−3.69
*FADS1*	fatty acid desaturase 1	0.001	−1.81
*FADS2*	fatty acid desaturase 2	0.0006	−1.84
*GLEAN_10000268*	GLEAN_10000268	0.001	−1.91
*GUCA2B*	guanylate cyclase activator 2B (uroguanylin)	0.001	−2.11
*HMGCS1*	3-hydroxy-3-methylglutaryl-CoA synthase 1 (soluble)	0.001	−2.21
*IFI6*	interferon, alpha-inducible protein 6	0.001	−2.27
*KCTD14*	potassium channel tetramerisation domain containing 14	0.0006	−1.82
*LOC100125623*	period 3	0.001	−2.09
*LOC105614373*	cytochrome P450 2B11-like	0.0006	1.57
*MID1IP1*	MID1 interacting protein 1	0.001	−2.49
*MTHFD1L*	monofunctional C1-tetrahydrofolate synthase, mitochondrial-like	0.0006	−1.63
*OSGIN1*	oxidative stress induced growth inhibitor 1	0.001	−2.15
*PPP1R3B*	protein phosphatase 1, regulatory subunit 3B	0.001	−2.3
*SCD*	stearoyl-CoA desaturase (delta-9-desaturase)	0.001	−4.44
*SLC19A1*	solute carrier family 19 (folate transporter), member 1	0.0007	1.4
*THRSP*	thyroid hormone responsive	0.0006	−6.86
*ZNF564*	zinc finger protein 564	0.001	−2.52

^1^CON: weaned lambs fed commercial concentrates; ALF: unweaned grazing alfalfa lambs.

^2^q value: significance level.

^3^FC, fold-change.

Regarding SF, when ALF treatment was compared with CON, only four genes were differentially expressed with an FDR = 0.051, and all of them were upregulated in the ALF group (**Table 5**).

**Table 5:** Significant differentially expressed genes in subcutaneous fat and fold change in ALF–CON^1^ contrast.

Gene symbol	Gene Name	q value^2^	FC^3^
*CLDN12*	claudin 12	0.01	1.26
*LOC101121796*	cytochrome P450 2B19-like	0.047	1.70
*METTL1*	methyltransferase like 1	0.047	1.33
*GPN2*	GPN-loop GTPase 2	0.047	1.31

^1^CON, weaned lambs fed commercial concentrates; ALF, unweaned grazing alfalfa lambs.

^2^q value: significance level.

^3^FC, fold-change.

#### Treatment-Dependent Multivariate Analysis Results of Gene Expression in *Longissimus thoracis* Muscle, Liver and Subcutaneous Fat

**Figure 2:**
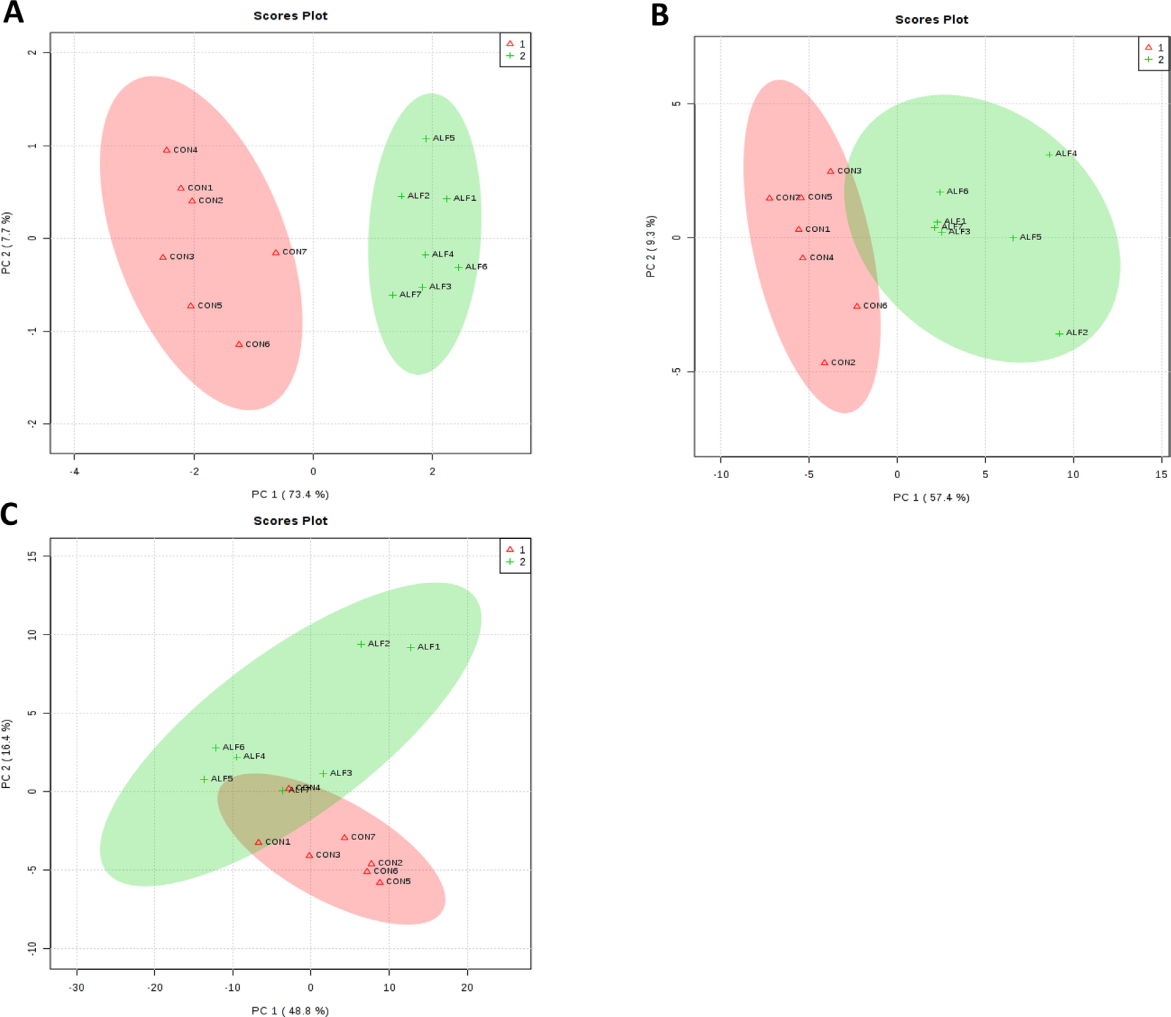
Principal components analysis (PCA) based on gene expression profile data for the **(A)** LT muscle **(B)** liver, and **(C)** subcutaneous fat. Principal components analysis scores plots discriminating between the LT muscle of lambs fed concentrate CON (+) and Alfalfa (triangle).

Principal component (PC) analysis of the complete set showed that the first two PCs covered 81.1% of the observed variance of the sample set in LT muscle (**Figure 2A**). The clusters corresponding to gene expression profiles from ALF and CON groups were clearly separated from each other. Very similar results were obtained in the liver (**Figure 2B**), but in SF, this separation was less clear (**Figure 2C**).

#### Hierarchical Clustering Analysis (HCA) in *Longissimus Thoracis* Muscle and Liver

**Figure 3:**
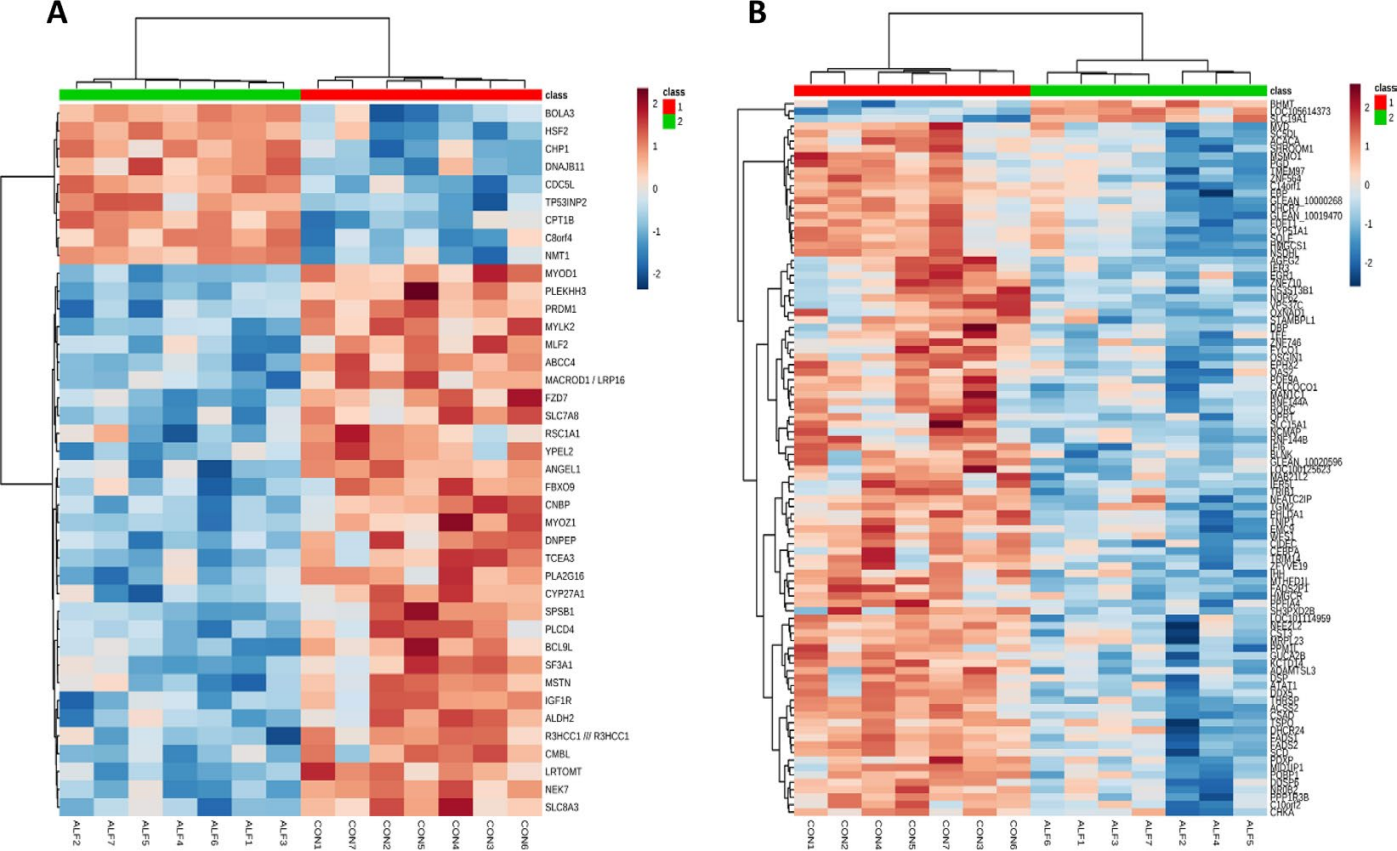
Hierarchical clustering analysis of significant gene expression in the **(A)** LT muscle and **(B)** liver of CON lambs (1) and ALF lambs (2). Cells are colored based on the measured signal intensity. Dark brown represents high gene expression levels, blue indicates low signal intensity, and gray cells represent the intermediate level.

Hierarchical clustering analysis for gene expression was performed using all genes and only the significant genes for each tissue. Because only four genes were significant in SF, the results of cluster analysis are not included. The results of HCA using only the significant genes for LT muscle and liver are presented in **Figure 3**. The expression profile of these genes was able to cluster and correctly classify the samples within their corresponding group. The heatmap shows the presence of two different clusters in both tissues. These two clusters clearly distinguished the ALF group from the CON group, as both groups showed very different gene expression patterns. For example, in LT muscle, the genes *BOLA*, *HSF2*, *CHP1*, *DNAJB11*, *CDC5L*, *TP53INP2*, *CPT1B*, *C8ORF4*, and *NMT1* were upregulated in the ALF group. Furthermore, a second cluster including the rest of the genes was found to be downregulated in the ALF group (**Figure 3A**). In the liver, the genes *BHMT*, *LOC105614373* and *SLC19A1* were upregulated in the ALF group, and a second cluster, including the remaining genes, was downregulated in the ALF group (**Figure 3B**).

### Functional Clustering Annotation

#### *Longissimus thoracis* Muscle

**Figure 4:**
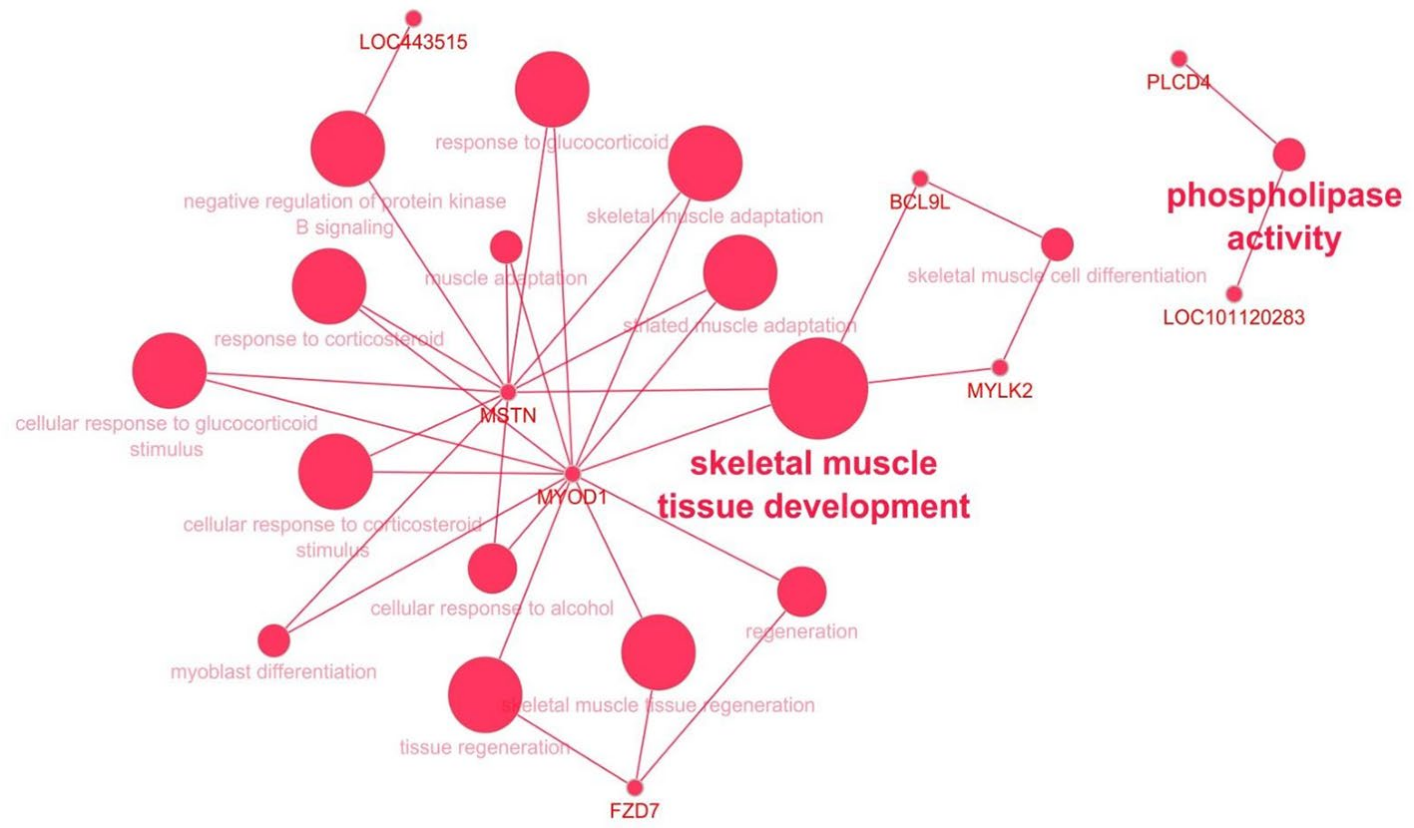
The biological role of up- (green) and downregulated (red) genes in the LT muscle of ALF animals visualized with ClueGO. The size of the nodes reflects the statistical significance of the term.

To gain insight into the biological processes that are regulated differentially between dietary treatments, we performed enrichment analyses using DAVID and ClueGo. The results of DAVID functional annotation clustering (FAC) revealed that the most enriched functional clusters were associated with “lipid and catabolic processes” (*CPT1B*, *PLA2G16*, *SPSB1*, *LRTOMT*, *PLCD4*, *FBXO9*, *CNBP* and *CYP27A1*) and 14 genes related to “muscle development” (*ALDH2*, *ANK3*, *CPT1B*, *FZD7*, *HSF2*, *IGF1R*, *LRTOMT*, *MSTN*, *MYLK2*, *MYOD1*, *MYOZ1*, *NMT1*, *PRDM1* and *RSC1*) (**Supplementary Table 5**). All these genes were downregulated in the ALF group except *CPT1B*, *HSF2* and *NMT1*, although the confident enrichment scores were less than 1.3 in both cases. The biological roles of downregulated genes in LT muscle were also visualized with ClueGO (**Figure 4**). The size of the nodes reflects the statistical significance of the term. The most enriched biological process was that of “skeletal muscle tissue development” with four genes, *MSTN*, *MYLK2*, *MYOD1* and *BCL9L.*

#### Liver

**Figure 5:**
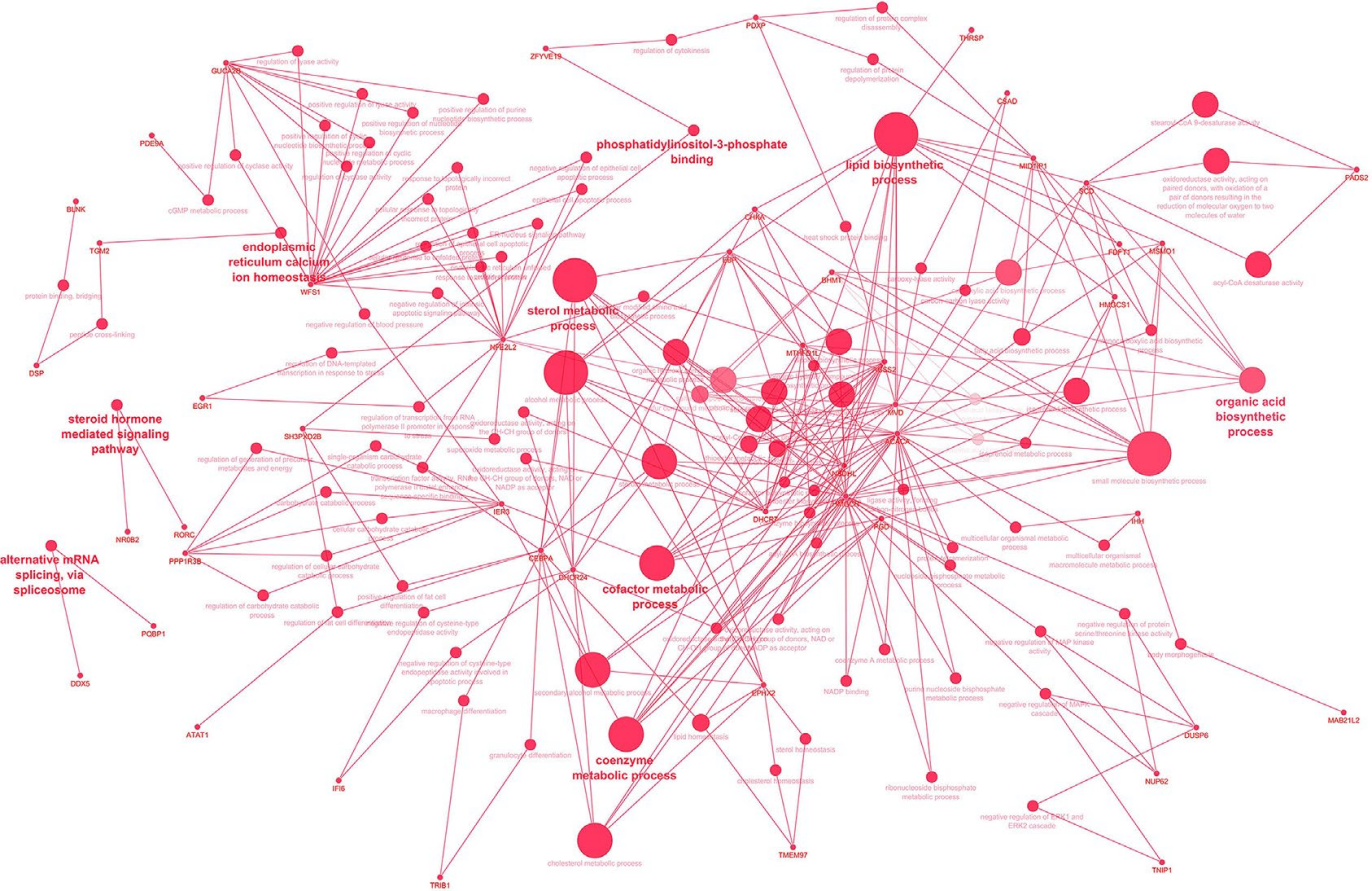
The biological role of up- (green) and downregulated (red) genes in ALF animals visualized with ClueGO in the liver. The size of the nodes reflects the statistical significance of the term.

The results of DAVID revealed two major gene clusters associated with “sterol biosynthesis” (*EBP*, *MVD*, *HMGCR*, *CYP51A1*, *HMGCS1*, *NR0B2*, *C14ORF1*, *FDFT1*, *SQLE*, *DHCR7*, *SC5DL*, *DHCR24*, and *NSDHL*), “lipid biosynthetic process” (*ACACA*, *CYP51A1*, *FADS1*, *FADS2*, *SCD* and *SC5DL*), and “cholesterol metabolic process” (*EBP*, *MVD*, *HMGCR*, *CYP51A1*, *SQLE*, *DHCR7*, *HMGCS1*, *NR0B2*, *DHCR24*, *FDFT1*, and *NSDHL*) (**Supplementary Table 6**). Similar results were obtained with ClueGo, where the most enriched biological processes were “fatty acid biosynthetic process,” “sterol metabolic process,” “cofactor metabolic processes” and “coenzyme metabolic processes” (**Figure 5**). These genes were all downregulated in ALF treatment.

#### Subcutaneous Fat

Only 4 genes were significant in SF, and no cluster was found with DAVID FAC.

### Validation of Microarray Results Using qPCR

The gene set selected to validate the microarray results by qPCR included the following 15 genes: *CPT1B*, *MYOD1*, *MSTN*, *ABCC4*, *IGF1R* and *PLA2G16* for LT muscle; *FADS1*, *FADS2*, *ACACA*, *SCD*, *SQLE*, *IER3*, *SLC19A1* and *THRSP* for liver; and *METTL1* for SF. The genes were selected because they were significantly differentially expressed between groups. The expression of these genes using microarray technology and qPCR is shown in **Table 6**. The housekeeping genes *GUSB* and *YWHAZ* were used to normalize the results for LT muscle and SF. In the liver, five candidate reference genes were tested, and the most stable genes exhibiting the lowest expression stability value (M) were *RPL37* (M = 0.182), *GUSB* (M = 0.226), and *RPL19* (M = 0.296). The three reference genes were more stable than the GOIs. The magnitude of the fold change obtained by microarray and qPCR was slightly different in some instances, but the qPCR results demonstrated a similar trend compared with the microarray results of these genes (**Table 6**).

**Table 6:** Real-time PCR confirmation of the microarray results. Gene expression changes in LT muscle, liver and subcutaneous fat in ALF vs CON comparison, and the fold change (FC) obtained with microarray and qPCR data.

Genes	Microarray	qPCR
**LT muscle**		
*CPT1B*	1.47**	1.15
*MYOD1*	−2.77**	−2.03*
*MSTN*	−2.43**	−5.73**
*ABCC4*	−1.42**	−1.89†
*IGF1R*	−1.19**	−1.79
*PLA2G16*	−1.36**	−2.27**
**Subcutaneous fat**		
*METTL1*	1.33*	1.55
**Liver**		
*FADS1*	−1.81**	−1.65
*FADS2*	−1.82**	−2.31*
*ACACA*	−1.99**	−1.52
*SCD*	−4.43**	−3.78*
*SQLE*	−1.42**	−1.89†
*IER3*	−1.95**	−1.45
*SLC19A1*	1.41**	1.21
*THRSP*	-6.86**	−6.13**

*P < 0.05, ** P < 0.01 and †0.05 < P < 0.10.

## Discussion

In this study, we investigated the fatty acid profile and gene expression using a microarray in the LT muscle, liver and SF of lambs fed concentrate or alfalfa. As expected, ALF animals contained greater CLAs and a greater proportion of n−3 PUFAs in muscle, such as linolenic acid (C18:3 n−3), EPA (C20:5 n−3), docosapentaenoic acid (C22:5 n−3), and DHA (C22:6 n−3), when compared with levels in the CON group. Many studies have reported the impact of grazing on the fatty acid profile in meat lambs, particularly the fatty acids of the n−3 series (Fisher et al., 2000; Dervishi et al., 2010; Vasta et al., 2012). Zhang et al. (2017) suggested that specific compounds in the diet can be transferred to the meat. In our experiment, the fatty acid composition, especially that of the n−3 series, and α-tocopherol are probably a reflection of diet composition. Suckling lambs are functionally non-ruminants, and their meat FA profile should reflect the FA profile of the suckled milk (Napolitano et al., 2002; Valvo et al., 2005). Thus, grazing dams is an advisable alternative to increase PUFAs in the suckling lamb meat because fresh pasture has a high concentration of linolenic acid (C18:3n−3), which increases the contents of vaccenic acid (C18:1t-11), conjugated linoleic acid isomers (CLA), and n−3 PUFA in milk compared with diets comprising concentrate or preserved forage (Nudda et al., 2005; Joy et al., 2012). The high value of C18:3n−3 in ALF lamb meat could be due to the C18:3n−3 provided by pasture that, as they are not yet fully weaned, is not bio-hydrogenated by rumen microbiota. Moreover, the relatively low effectiveness of milk in affecting meat fatty acid composition, could explain the slightly difference between CON and ALF in CLA and VA. Therefore, lambs that were allowed to graze resulted in a meat fatty acid profile that is richer in fatty acids of the n−3 series, mainly due to the dam’s milk that were grazing continuously during lactation in alfalfa pastures. According to Álvarez-Rodriguez et al. (2018) dietary alfalfa but not milk supply improved CLA, and n−3 PUFAs contents in lamb meat. The FA composition of ALF lambs was more related to ewe’s milk than to fresh forage (Dervishi et al., 2010). Previous studies have shown that grazing increases the PUFA content in milk, particularly linolenic acid (C18:3n−3), while concentrates modify rumen retention time of the feed, increase linoleic acid (C18:2n−6) intake, and alter biohydrogenation pathways toward lower n−3 PUFA and CLA contents, leading to lower contents of these compounds in the milk (Elgersma, 2015). In addition, these animals had greater α-tocopherol in muscle and plasma. Vitamin E is a powerful fat-soluble antioxidant that plays important roles in scavenging free radicals and neurologic function (Wang and Quinn, 2000; Traber and Atkinson, 2007). In this study, we found that lipid oxidation was lower in ALF lambs on day 7 of display when compared with the levels in CON lambs. These results are in concordance with previous studies in which we reported that the addition of vitamin E to the diet increased the α-tocopherol muscle content and drastically diminished the lipid oxidation of meat (Kasapidou et al., 2012; González-Calvo et al., 2015b; Ponnampalam et al., 2017).

Moreover, we investigated how the feeding system impacted gene expression in LT muscle, liver and SF in both treatment groups. Indeed, we found that both groups differed in their gene expression profile, mainly in LT muscle and liver, with the greatest impact in liver. It has been reported that dietary intervention can lead to major changes in gene expression in muscle and liver (Dervishi et al., 2011; Cui et al, 2018). In the ALF group, the most enriched biological processes in LT muscle were skeletal muscle tissue development (*MYOD1*, *MYLK2* and *MSTN*) (**Figure 4** and **Supplementary Table 5**). These genes were downregulated in the ALF group, with *MYOD1* and *MSTN* being the most downregulated genes (the lowest FC). The yield of saleable meat and meat quality, and therefore the profitability for livestock operations, are greatly influenced by growth during the postnatal period. Therefore, the identification of genes that play a role in muscle growth in sheep is an important step for improving sheep meat production by selection. In this regard, in livestock species, *MYOD1* and *MSTN* are considered candidate genes for meat quality and carcass traits (Ibeagha-Awemu et al., 2008; Bhuiyan et al., 2009). *MYOD1* regulates muscle cell differentiation, growth, and development and is also involved in muscle regeneration (Kitzmann et al., 1998). For example, polymorphisms of *MYOD1* have been associated with weight, several muscle fiber characteristics, the loin eye area and lightness in yak populations, pork and cattle (Chu et al., 2012; Lee et al., 2012; Du et al., 2013). In addition, low *MYOD1* expression levels were related to low Warner–Bratzler shear force measured in the *longissimus dorsi* muscle of beef (Tizioto et al., 2014) and thus with greater tenderness. In sheep, a positive correlation between *MYOD1* expression and cold carcass yield was found (Lôbo et al., 2012). The authors proposed that animals with a higher expression of *MYOD1* were more efficient during postnatal growth and had a greater *longissimus* dorsi weight and a better cold and hot carcass yield. In our study, we did not observe differences in slaughter weight or average daily gain between the ALF and CON groups. These discrepancies may be due to different slaughter ages among both studies. We sacrificed our animals at 67–72 days, and other studies compared heavy lambs (at an average of 200 days) fed either concentrate or limited grazing (Lôbo et al., 2012), whereas in the present study, grazing lambs had free access to forage, concentrate, and dam’s milk. Despite the different results, further investigation into the role of *MYOD1* in sheep carcass and meat quality traits in sheep is necessary for effective marker assisted selection. Another gene that was downregulated in the ALF group was *MSTN.* Myostatin is an extracellular cytokine that is mostly expressed in skeletal muscles and is known to play a crucial role in the negative regulation of muscle mass (Elkina et al., 2011). This effect is due to an increase in both muscle fiber number (hyperplasia) and mass (hypertrophy). For instance, Ji et al. (1998) demonstrated that myostatin expression in skeletal muscle peaks prenatally and that greater expression during the prenatal period is associated with low birth weight in pigs. Mutations in the myostatin gene with functional inactivation in beef cattle increase the muscle mass in the double-muscled phenotype and lead to smaller adipocytes and fewer fat islands in muscle (Cassar-Malek et al., 2007). In addition, in different cattle breeds, mutations in *MSTN* have been associated with significant reductions in the shear force and a decrease in total collagen content (Ngapo et al., 2002; Lines et al., 2009). Moreover, mutations in *MSTN* in sheep were associated with muscling and reduced intramuscular fat (Kijas et al., 2007) and an increased percentage of fast glycolytic myofibers (Laville et al., 2004). In our experiment, the ALF group showed downregulated *MYOD1* and *MSTN* genes, which may be beneficial for increasing meat tenderness and cold carcass yield in heavier animals. However, the simultaneous downregulation of the *MYOD1* and *MSTN* genes in ALF group might determine an opposite effect about animal’s performance, thus justifying the lack in different performance between two groups.

The results of the functional analysis showed that genes related to catabolic and lipid processes in LT muscle were downregulated (*PLA2G16*, *SPSB1*, *LRTOMT*, *PLCD4*, *FBXO9*, *CNBP* and *CYP27A1*), except for *CPT1B*, which was upregulated in the ALF group (**Supplementary Table 5**). Carnitine palmitoyl transferase I (M-CPT 1), codified by the *CPT1B* gene, is part of the mitochondrial transport system and is a key enzyme in the control of long-chain fatty acid oxidation (Bartelds et al., 2004). These results are in agreement with those previously obtained by Dervishi et al. (2011, 2012) in which grazing systems promoted higher levels of *CPT1B* gene expression in the *semitendinosus* muscle and mammary gland. As reported by Dervishi et al. (2010), concentrate feeding promotes the upregulation of genes related to adipogenesis, whereas the grazing system promotes higher levels of genes implicated in fatty acid oxidation.

The impact of feeding system was more pronounced on liver gene expression, where 96 genes were significantly changed compared to that in LT muscle (41 genes) and SF (4 genes). The major sites of fatty acid synthesis are adipose tissue and the liver. However, the results for gene expression in these three different tissues suggest that in young lambs, the major site of lipid metabolism is the liver rather than subcutaneous fat.

**Figure 6:**
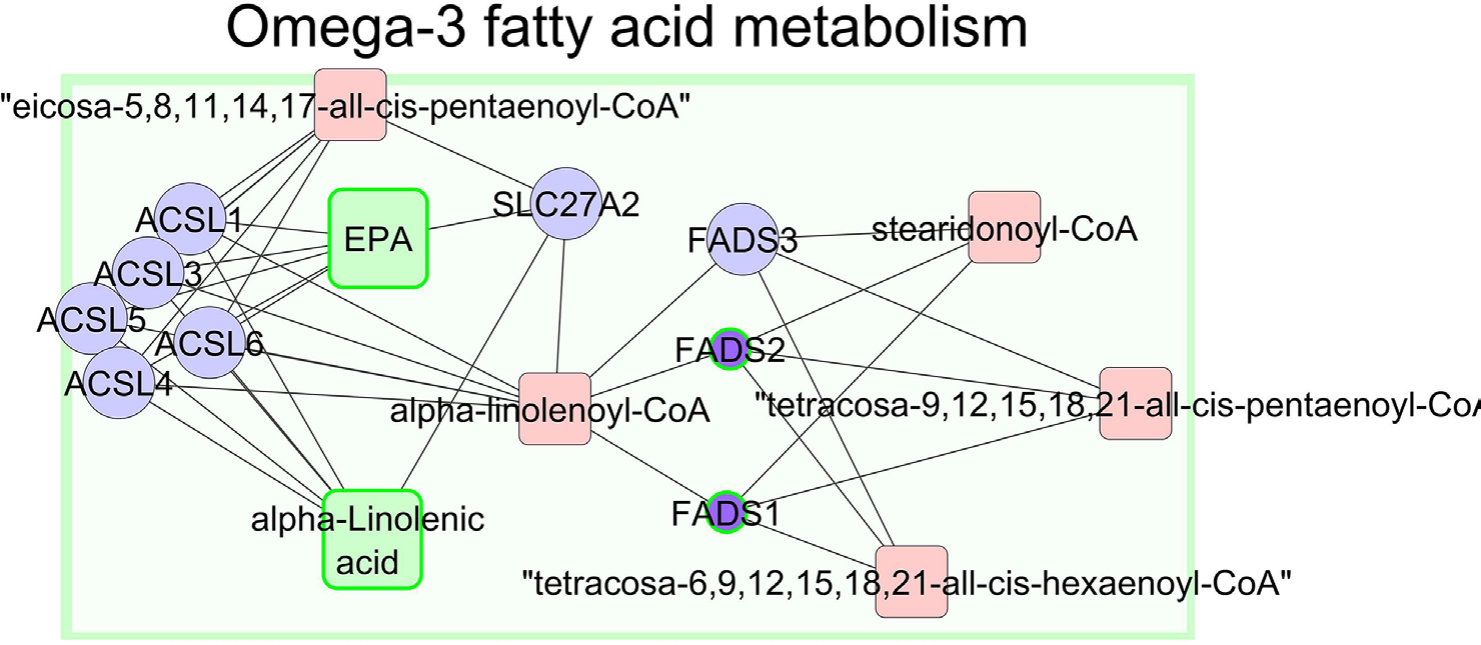
The network of metabolites and genes involved in omega-3 fatty acid metabolism in ALF lambs. Significant metabolites with experimental data are shown in green squares, and significant genes with experimental data are shown in purple circles with green borders. The size of the nodes represents the direction of the change. A small purple circle with a green border indicates downregulated genes, and large green square nodes point to upregulated compounds.

We attempted to link the significant fatty acids in muscle and metabolites in plasma with the results of gene expression to obtain a better understanding of the underlying metabolic processes associated with different feeding systems. The relationship between n−3 FAs in muscle and gene expression in liver mapped 30 significant genes (out of 92) and 9 significant compounds (out of 13) to KEGG IDs. A compound-gene network was generated (**Figure 6** and **Supplementary Figure 2**). In addition, this approach helped us to identify genes related to enriched biological processes and certain desired outcomes, for example, n−3 PUFA series that are desirable regarding human health (Simopoulos, 2008; Cabo et al., 2012; Liu and Ma, 2014). Indeed, the animals grazing alfalfa had a greater content of fatty acids of the n−3 series such as linolenic acid (C18:3 n−3), EPA (C20:5 n−3), docosapentaenoic acid (C22:5 n−3), and DHA (C22:6 n−3) in LT muscle and lower expression of *FADS1* and *FADS2* in liver (**Figure 6**). It is worth mentioning that *FADS1* and *FADS2* in the “fatty acid biosynthetic process” cluster are key genes in the metabolism of n−3 PUFA series. The proteins encoded by these genes (*FADS1* and *FADS2*) are members of the fatty acid desaturase (FADS) gene family. Desaturase enzymes regulate the unsaturation of fatty acids through the introduction of double bonds between defined carbons of the fatty acyl chain (Nakamura and Nara, 2004). In addition, these animals were characterized by a decrease in the expression of genes related to cholesterol metabolism (*DHCR7*, *SC5DL*, *EBP*, *NSDHL*, *MTHFD1L*, and *CYP51A1*; **Supplementary Figure 2**).

Nutrition is an important strategy to alter gene expression and the fatty acid profile of meat. It has been widely reported that grazing animals have a greater content of the n−3 PUFA series in the serum, liver and muscle and a lower n−6:n−3 ratio, in agreement with the present study. Interestingly, we also found that the expression of two genes related to n−3 PUFA metabolism was downregulated in the livers of ALF animals. *Fatty acid desaturase 1* (*FADS1*) and *2* (*FADS2*) genes encode delta-5 and delta-6 desaturases, respectively, which are rate-limiting enzymes in the synthesis of polyunsaturated omega-3 and omega-6 FAs. Dietary FAs have been shown to regulate desaturase activity (Nakamura and Nara, 2004). Gene expression of both *FADS1* and *FADS2* is reduced by PUFAs in several hepatic models (Reardon et al., 2013; Cho et al., 1999a; Cho et al., 1999b). Furthermore, *FADS1* and *FADS2* gene expression was reduced by EPA and AA in 3T3‐L1 adipocytes (Ralston et al., 2015). In our study we found that ALF lambs have greater amount of EPA in their muscle mainly because of their diet. ALF lambs ingested diets rich in PUFAs (fresh alfalfa, and mainly dams’ milk), which in turn might have down-regulated *FADS1* and *FADS2* gene expression in liver. In support to our speculation da Costa et al. (2014) found that high levels of n−3 PUFA in cattle liver down-regulated the expression of the genes *FADS1* and *FADS2*.

The results found in the current study showed that ingesting diets richer in n−3 PUFA might have negative effects on the *de novo* synthesis of n−3 PUFA by the FADS1 and FADS2 enzymes. However, feeding diets poorer in n−3 PUFA can promote fatty acid desaturation, which makes these two genes attractive candidates for altering the content of PUFAs in meat, by looking for polymorphisms that may affect the functionality and efficiency of these enzymes and alter the fatty acid profile in lamb meat. Functional SNPs can provide an additional resource as a potential genetic markers in breeding programs. In this respect, in humans, numerous studies have consistently replicated the associations between polymorphisms in the *FADS1* and *FADS2* genes and the PUFA concentration (Corella and Ordovas, 2012). In porcine, a polymorphism in exon 3 of the pig *FADS2* has been associated with C20:4 and intramuscular fat (IMF) content (Renaville et al., 2013; Gol et al., 2018). In dairy cows, Ibeagha-Awemu et al. (2014) demonstrated positive associations between three SNPs within *FADS1* and *FADS2* with three milk PUFAs. Meanwhile, contradictory results have been reported in sheep. For example, a SNP in *FADS2* was significantly associated with intramuscular levels of EPA (C20:5n−3) and DHA (C22:6n−3) (Malau-Aduli et al., 2011), but in a different report, no SNP within the *FADS1* and *FADS2* gene regions was associated with lamb muscle n−3 levels (Knight et al., 2012). Our study further points to the importance of nutritional modulation of *FADS1* and *FADS2* gene expression and the fatty acid profile in sheep.

## Conclusion

Grazing lambs presented a higher content of CLA and n−3 PUFA series and showed a lower n−6/n−3 ratio, which is favorable with regard to current human health. The feeding system is the main factor affecting the fatty acid composition and gene expression in LT muscle and liver. The gene expression results in the three different tissues suggest that the major site of lipid metabolism is the liver rather than subcutaneous fat in young lambs of the Rasa Aragonesa breed. Gene expression of *FADS1* and *FADS2* plays an important role in the synthesis of n−3 PUFA series, which in turn makes these two genes attractive candidates to alter the content of PUFAs in meat. More studies will be necessary to elucidate the effects of the feeding system on *FADS1* and *FADS2* expression in other tissues of interest or to search for mutations or functional SNPs that may be used in the future as a tool to improve the fatty acid profile in lamb meat.

## Data Availability Statement

The datasets generated for this study can be found in NCBI Gene Expression Omnibus repository, GSE63774 (LT muscle and SF) and GSE125661 (liver).

## Ethics Statement

All Experimental Procedures, Including the Care of Animals and Euthanasia, Were Performed in Accordance With the Guidelines of the European Union and Spanish Regulations for the Use and Care of Animals in Research and Were Approved by the Animal Welfare Committee of the Centro De Investigación Y Tecnología Agroalimentaria (CITA) (Protocol Number 2009-01_MJT). in All Cases, Euthanasia Was Performed by Penetrating Captive Bolt Followed by Immediate Exsanguination.

## Author Contributions

LG-C, PS, and MB performed the experiments. MJ, MS, and JC designed the research and obtained funding for this research. ED, MS, MB, and JC wrote the paper. ED, LG-C, MS, MB, MJ, RM-H, JO, and JC analyzed the data. MJ provided animals. JC had primary responsibility for the final content. All of the authors contributed to the manuscript discussion. All of the authors read and approved the final manuscript.

## Funding

This study was supported by the Ministry of Economy and Competitiveness of Spain, the European Union Regional Development Funds (INIA RTA2012-080-00, INIA RZP2017-00001-00) and the Research Group Funds of Aragón Government (A14_17R).

## Conflict of Interest

The authors declare that the research was conducted in the absence of any commercial or financial relationships that could be construed as a potential conflict of interest.
