# Supplementary material for: Gene Expression and Fatty Acid Profiling in Longissimus thoracis Muscle, Subcutaneous Fat, and Liver of Light Lambs in Response to Concentrate or Alfalfa Grazing
Source: Front Genet. 2019 Oct 31;10:1070. doi: 10.3389/fgene.2019.01070 (PMC6834778; doi:10.3389/fgene.2019.01070)
Supplement: Supplementary file 1 [file DataSheet_1.docx]

**Supplementary Material**

**Supplementary Table 1.** Effect of the dietary treatment on slaughter age and weight, growth rate from birth to slaughter

|  | CON | ALF |  | SE | P-value |
| --- | --- | --- | --- | --- | --- |
| **Performance** |  |  |  |  |  |
| Slaughter Age (d) | 72 | 67 |  | 3.25 | ns |
| Slaughter Weight (kg) | 23.2 | 23.0 |  | 0.40 | ns |
| Average Daily Gain (g/d) | 278 | 277 |  | 11.1 | ns |

^1^CON: commercial concentrate; ALF: unweaned lambs grazing alfalfa plus commercial concentrate. SE: standard error; ns: not significant;

**Supplementary Table 2**. Primers forward and reverse used in RT-PCR.

| **PCR conditions**^1^ | | | | | | | |  |
| --- | --- | --- | --- | --- | --- | --- | --- | --- |
| **Symbol** | **Gene** | **Primers forward and reverse** | **Amplicon (bp)** | **AT** | **nM** | **E** | **R^2^** | **Function** |
| **LT Muscle and SF** | | | | | | | |  |
| *CPT1B* | Choline kinase beta; carnitine palmitoyltransferase 1B (muscle) | F:5´- tgttcaacaccactcgcatc -3´  R:5´- ctcgtagagccacagcttga -3´ | 116 | 60 | 600/600 | 1.99 | 0.99 | Carnitine O-palmitoyltransferase activity |
| *MYOD1* | Myogenic differentiation 1 | F:5´-aacactacagcggcgact-3´  R:5´-cggtcgtagcagttccgt-3´ | 91 | 60 | 600/600 | 1.98 | 0.99 | Skeletal muscle tissue development |
| *MSTN* | Myostatin | F:5’- accttcccagaaccaggaga-3’  R:5’- cgagattctgtggagtgctca -3’ | 122 | 59 | 900/900 | 2.00 | 0.99 | Skeletal muscle tissue development |
| *ABCC4* | ATP-binding cassette, sub-family C (CFTR/MRP), member 4 | F: 5´-ccgtgagaaatttgcccactg-3´  R: 5´- gcaaaacatacggctcatcat-3´ | 128 | 60 | 900/900 | 2.00 | 0.99 | Organic anion pump relevant to cellular detoxification |
| *IGF1R* | Insulin-like growth factor 1 receptor | F: 5´-ccaaggcctgagaactccat-3´  R: 5´- ctggacacacattcccgct-3´ | 122 | 60 | 900/900 | 2.00 | 0.98 | Cell growth and survival control |
| *PLA2G16* | Phospholipase A2, group XVI | F: 5´-cgagaactgtgagcacttcg-3´  F: 5´-ctcccgcgatgccaactg-3´ | 95 | 60 | 900/900 | 1.98 | 0.99 | Phospholipase A1 and A2 activity |
| *METTL1* | Methyltransferase like 1 | F:5´-ggagctgcatgagtggatgt-3´  R:5´- tgcccagatgtcccacaatg-3´ | 104 | 60 | 600/600 | 1.97 | 0.99 | Catalyzes the formation of N(7)-methylguanine at position 46 (m7G46) in tRNA. |
| *GUSB* | β-glucuronidase | F:5´-gcttcgagcagcagtggta-3´  R:5´-cacgtcgttgaagctggac-3´ | 86 | 60 | 600/600 | 1.99 | 0.99 | Housekeeping |
| *YWHAZ* | Tyrosine 3-monooxygenase/tryptophan 5-monooxygenase activation protein | F:5´- tgtaggagcccgtaggtcatct-3´  R:5´ - ttctctctgtattctcgagccatct-3 | 102 | 60 | 400/400 | 1.99 | 0.98 | Housekeeping |
| **Liver** | | | | | | | |  |
| *FADS1* | Fatty acid desaturase 1 | F:5´- atgcaacgtccacaacaagtcag-3´  R:5´ - gggagccactttgtggtaat-3 | 115 | 59 | 600/600 | 2.00 | 0.99 | Involved in biosynthesis of polyunsaturated fatty acids (PUFA). |
| *FADS2* | Fatty acid desaturase 2 | F:5´- gttcagtggggacctcaact-3´  R:5´ - tcatcaatgccgtgcttg-3 | 120 | 60 | 900/900 | 2.04 | 0.99 | Involved in biosynthesis of polyunsaturated fatty acids (PUFA).. |
| *ACACA* | Acetyl-CoA carboxylase alpha | F- 5´tgaaggatgtggatgatgg- 3´  R- 5´cgcgttgttgacttttctga- 3´ | 110 | 59 | 900/900 | 1.98 | 0.99 | Catalyzes the carboxylation of acetyl-CoA to malonyl-CoA, the rate-limiting step in fatty acid synthesis. |
| *SCD* | Stearoyl-CoA desaturase | F- 5´cccagctgtcagagaaaagg- 3´  R- 5´gatgaagcacaacagcagga- 3´ | 115 | 60 | 900/900 | 1.99 | 0.99 | Involved in fatty acid biosynthesis, primarily the synthesis of oleic acid. |
| *SQLE* | Squalene epoxidase | F:5´- aatgtgttgcaggtccggtt-3´  R:5´ - tagactgcgacgccaaagaa-3 | 88 | 60 | 900/900 | 2.03 | 0.99 | Catalyzes the first oxygenation step in sterol biosynthesis and is thought to be one of the rate-limiting enzymes in this pathway. |
| *SLC19A1* | Solute carrier family 19 (folate transporter), member 1 | F:5´- accatcatcaccctcatcgt-3´  R:5´ - gaagtaggcggcgaagagta-3 | 108 | 60 | 400/400 | 2.08 | 0.98 | Transporter for the intake of folate. |
| *THRSP* | Thyroid hormone responsive | F:5´- gcacttcgctagccttcatc-3´  R:5´ - ctacatggcctgtcccattt-3 | 97 | 60 | 900/900 | 2.04 | 0.99 | Plays a role in the regulation of lipogenesis, being important for the biosynthesis of triglycerides with medium-length fatty acid chains. |
| *GUSB* | β-glucuronidase | F:5´-gcttcgagcagcagtggta-3´  R:5´-cacgtcgttgaagctggac-3´ | 86 | 59 | 400/400 | 2.06 | 0.99 | Housekeeping |
| *RPL19* | Ribosomal protein L19 | F-5´caactcccgccagcagat-3´  R-5´ccgggaatggacatgtcaca-3´ | 76 | 59 | 600/600 | 2.08 | 0.99 | Housekeeping |
| *RPL37* | Ribosomal protein L37 | F-5´gaagtcgacctgtggcaagt-3´  R-5´ctcattcgaccagtcccagt-3´ | 105 | 59 | 600/600 | 2.01 | 0.98 | Housekeeping |

^1^Real-time PCR conditions: annealing temperature (AT), primer concentrations (nM), E: primer efficiency and R^2^: correlation coefficient.

**Supplementary Table 3**. Type III test of the fixed effects in blood parameters

| **Efect** | **Treatment** | **Day** | **Treatment*Day** |
| --- | --- | --- | --- |
| α-tocopherol | <.0001 | 0.0003 | 0.0041 |
| cholesterol | 0.056 | <.0001 | 0.0389 |
| HDL- cholesterol | 0.003 | <.0001 | 0.0009 |
| LDL- cholesterol | 0.081 | <.0001 | 0.0117 |
| TG | 0.006 | 0.4944 | 0.0849 |

**Supplementary Table 4**. Effect of the treatment (LSMeans and standard error) on meat quality traits in Longissimus thoracis muscle

| **Treatment^1^** | | | | | |  |
| --- | --- | --- | --- | --- | --- | --- |
|  | **CON** |  | **ALF** |  | **P-value Tukey** | |
| IMF (% fresh matter) | 2.03(0.19) |  | 1.74(0.19) |  | 0.31 | |
| TBARS (7 d)^1^ | 1.62 (0.13) |  | 0.65 (0.15) |  | 0.004 | |
| K/S_572/525_ (7 d)^2^ | 0.93 (0.04) |  | 1.01 (0.03) |  | 0.93 | |
| Lightness (L*) | 45.32 (0.97) |  | 41.53 (0.91) |  | 0.11 | |
| Redness (a*) | 10.12 (0.74) |  | 11.47 (0.68) |  | 0.86 | |
| Yellowness (b*) | 8.21 (0.61) |  | 6.60 (0.58) |  | 0.56 | |
| Hue angle (H_ab_) | 40.63 (3.75) |  | 29.59 (3.59) |  | 0.45 | |
| Chroma (C* _ab_) | 13.18 (0.53) |  | 13.33 (0.49) |  | 1 | |

^1^CON: commercial concentrate; ALF: unweaned grazed on alfalfa pasture ^1^TBARS: mg of malonaldehyde per kg of L. Thoracis muscle at 7 days of display; ^2^K/S_572/525_: ratio of metmyoglobin formation of LT muscle at 7 days of display.

**Supplementary Table 5.** DAVID Functional Annotation Clustering^1^ of SAM genes in ALF vs. CON^2^ in LT muscle.

| **Annotation Cluster 1 Enrichment Score: 1.0880364970730936** | | | | | |
| --- | --- | --- | --- | --- | --- |
| **Category** | **Term** | **Count** | **%** | **P value** | **Genes** |
| GOTERM_BP_FAT | GO:0016042~lipid catabolic process | 3 | 0,74 | 0,06 | CPT1B, PLA2G16, PLCD4 |
| GOTERM_BP_ALL | GO:0006629~lipid metabolic process | 5 | 1,23 | 0,10 | CPT1B, CNBP, PLA2G16, CYP27A1, PLCD4 |
| GOTERM_BP_ALL | GO:0009056~catabolic process | 6 | 1,47 | 0,14 | CPT1B, PLA2G16, SPSB1, LRTOMT, PLCD4, FBXO9 |
| **Annotation Cluster 2; Enrichment Score: 0.9192169443966314** | | | | | |
| **Category** | **Term** | **Count** | **%** | **P value** | **Genes** |
| GOTERM_BP_FAT | GO:0007517~muscle organ development | 4 | 0,98 | 0,01 | CPT1B, MYOD1, MYLK2, MSTN |
| GOTERM_BP_ALL | GO:0032502~developmental process | 12 | 2,95 | 0,07 | IGF1R, CPT1B, MYOD1, NMT1, ANK3, ALDH2, MYLK2, MSTN, LRTOMT, MYOZ1, PRDM1, FZD7 |
| GOTERM_BP_ALL | GO:0048513~organ development | 8 | 1,97 | 0,08 | IGF1R, CPT1B, MYOD1, ALDH2, MYLK2, MSTN, LRTOMT, PRDM1 |
| GOTERM_BP_ALL | GO:0007275~multicellular organismal development | 11 | 2,70 | 0,08 | IGF1R, CPT1B, MYOD1, NMT1, ANK3, ALDH2, MYLK2, MSTN, LRTOMT, PRDM1, FZD7 |
| GOTERM_BP_ALL | GO:0048856~anatomical structure development | 10 | 2,46 | 0,09 | IGF1R, CPT1B, MYOD1, ANK3, ALDH2, MYLK2, MSTN, LRTOMT, MYOZ1, PRDM1 |
| GOTERM_BP_ALL | GO:0048731~system development | 9 | 2,21 | 0,13 | IGF1R, CPT1B, MYOD1, ANK3, ALDH2, MYLK2, MSTN, LRTOMT, PRDM1 |
| GOTERM_BP_ALL | GO:0032501~multicellular organismal process | 13 | 3,19 | 0,20 | MYOD1, CPT1B, MSTN, MYLK2, LRTOMT, FZD7, NMT1, IGF1R, HSF2, ANK3, ALDH2, PRDM1, RSC1A1 |
| GOTERM_BP_ALL | GO:0009888~tissue development | 3 | 0,74 | 0,43 | MYOD1, MYLK2, MSTN |
| GOTERM_BP_ALL | GO:0048518~positive regulation of biological process | 6 | 1,47 | 0,47 | IGF1R, MYOD1, CNBP, MYLK2, MSTN, PRDM1 |
| GOTERM_BP_ALL | GO:0048522~positive regulation of cellular process | 5 | 1,23 | 0,59 | IGF1R, MYOD1, CNBP, MYLK2, MSTN |
| GOTERM_BP_ALL | GO:0051239~regulation of multicellular organismal process | 3 | 0.74 | 0.62 | MYOD1, MYLK2, MSTN |

^1^Only the 2 most enrichment clusters are shown

^2^ CON: weaned lambs fed commercial concentrates; ALF: unweaned grazing alfalfa lambs

**Supplementary Table 6.** DAVID Functional Annotation Clustering of SAM genes^1^ in ALF vs CON^2^ in the liver.

| **Annotation Cluster 1 Enrichment Score: 5.822** | | | | | | |
| --- | --- | --- | --- | --- | --- | --- |
| **Category** | **Term** | **Nº genes** | **%** | | **P value** | **Genes** |
| GOTERM_BP_FAT | GO:0016126~sterol biosynthetic process | 12 | 14,11 | | 2,63E-17 | EBP, MVD, HMGCR, CYP51A1, SQLE, DHCR7, HMGCS1, C14ORF1, SC5DL, DHCR24, FDFT1, NSDHL |
| GOTERM_BP_FAT | GO:0016125~sterol metabolic process | 13 | 15,29 | | 2,72E-13 | EBP, MVD, HMGCR, CYP51A1, HMGCS1, NR0B2, C14ORF1, FDFT1, SQLE, DHCR7, SC5DL, DHCR24, NSDHL |
| GOTERM_BP_FAT | GO:0006695~cholesterol biosynthetic process | 9 | 10,58 | | 8,77E-13 | EBP, MVD, HMGCR, CYP51A1, DHCR7, HMGCS1, DHCR24, FDFT1, NSDHL |
| GOTERM_BP_FAT | GO:0006694~steroid biosynthetic process | 12 | 14,11 | | 1-09E-12 | EBP, MVD, HMGCR, CYP51A1, SQLE, DHCR7, HMGCS1, C14ORF1, SC5DL, DHCR24, FDFT1, NSDHL |
| GOTERM_BP_FAT | GO:0008610~lipid biosynthetic process | 18 | 21,17 | | 1,48E-12 | CHKA, EBP, MVD, HMGCR, CYP51A1, SCD, FADS1, HMGCS1, ACACA, FADS2, C14ORF1, ACSS2, FDFT1, SQLE, DHCR7, SC5DL, DHCR24, NSDHL |
| KEGG_PATHWAY | hsa00100:Steroid biosynthesis | 8 | 9,41 | | 1,34E-11 | EBP, CYP51A1, SQLE, DHCR7, SC5DL, DHCR24, FDFT1, NSDHL |
| GOTERM_BP_FAT | GO:0008202~steroid metabolic process | 14 | 16,47 | | 7,00E-11 | EBP, TSPO, MVD, HMGCR, CYP51A1, HMGCS1, NR0B2, C14ORF1, FDFT1, SQLE, DHCR7, SC5DL, DHCR24, NSDHL |
| GOTERM_BP_FAT | GO:0008203~cholesterol metabolic process | 11 | 12,94 | | 7,40E-11 | EBP, MVD, HMGCR, CYP51A1, SQLE, DHCR7, HMGCS1, NR0B2, DHCR24, FDFT1, NSDHL |
| GOTERM_BP_FAT | GO:0008299~isoprenoid biosynthetic process | 4 | 4,60 | | 1,74E-4 | MVD, HMGCR, HMGCS1, FDFT1 |
| GOTERM_BP_FAT | GO:0055114~oxidation reduction | 13 | 15,29 | | 1,89E-4 | HMGCR, CYP51A1, SCD, FADS1, PGD, FADS2, FDFT1, SQLE, DHCR7, OXNAD1, SC5DL, DHCR24, NSDHL |
| **Annotation Cluster 2 Enrichment Score: 3.593** | | | | | | |
| **Category** | **Term** | **Nº genes** | **%** | **P value** | | **Genes** |
| GOTERM_BP_FAT | GO:0006633~fatty acid biosynthetic process | 5 | 5,88 | 9,56E-4 | | FADS1, SCD, ACACA, FADS2, SC5DL |
| GOTERM_BP_FAT | GO:0016053~organic acid biosynthetic process | 6 | 7,06 | 1,68E-3 | | FADS1, SCD, BHMT, ACACA, FADS2, SC5DL |
| GOTERM_BP_FAT | GO:0046394~carboxylic acid biosynthetic process | 6 | 7,06 | 1,68E-3 | | FADS1, SCD, BHMT, ACACA, FADS2, SC5DL |
| GOTERM_BP_FAT | GO:0006631~fatty acid metabolic process | 6 | 7,06 | 4,81E-3 | | FADS1, SCD, EPHX2, ACACA, FADS2, SC5DL |

^1^Only the 2 most enrichment clusters are shown

^2^ CON: weaned lambs fed commercial concentrates; ALF: unweaned grazing alfalfa lambs

**Supplementary Figure 1.** Significant features identified by SAM in ALF-CON contrast in (a) LT muscle (b) liver, (c) SF. The green circles represent features that exceed the specified threshold.

a) b) c)


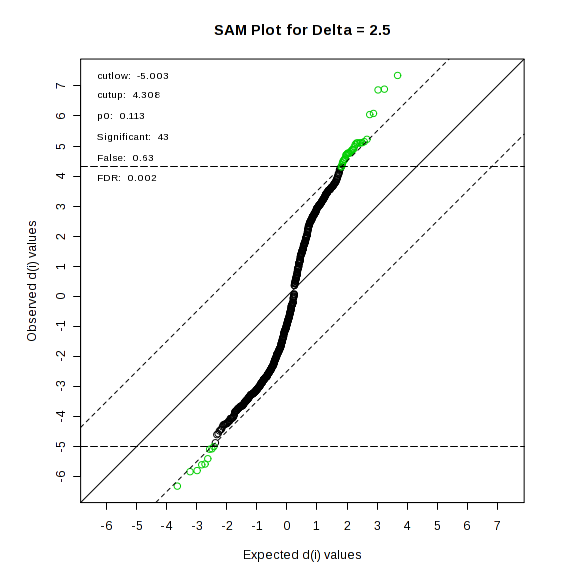

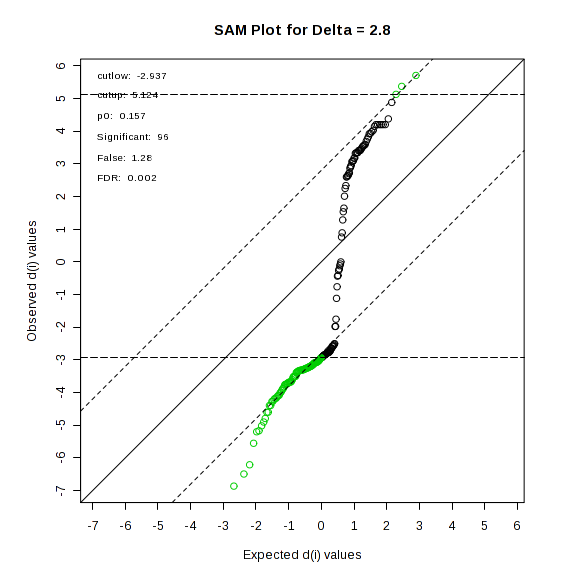

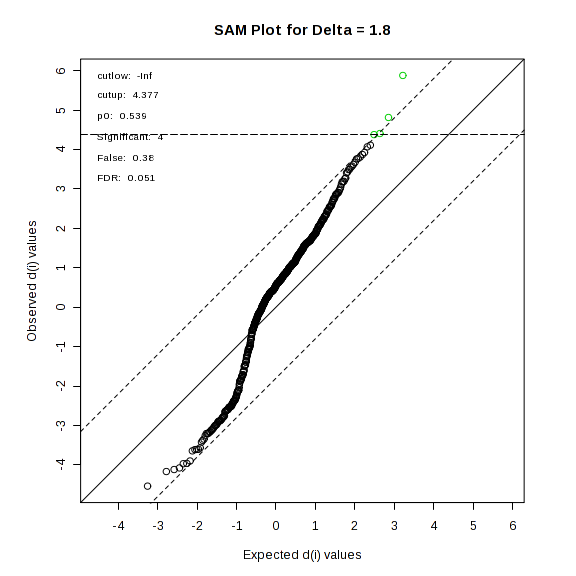


**Supplementary Figure 2**. The network of metabolites and genes involved in cholesterol metabolism in ALF lambs. Significant metabolites with experimental data are shown in green squares and significant genes with experimental data are shown in purple circle with green border. The size of the nodes represents the direction of the change. Small purple circle with green border point to down-regulated genes and big green square nodes points to up-regulated compounds

**
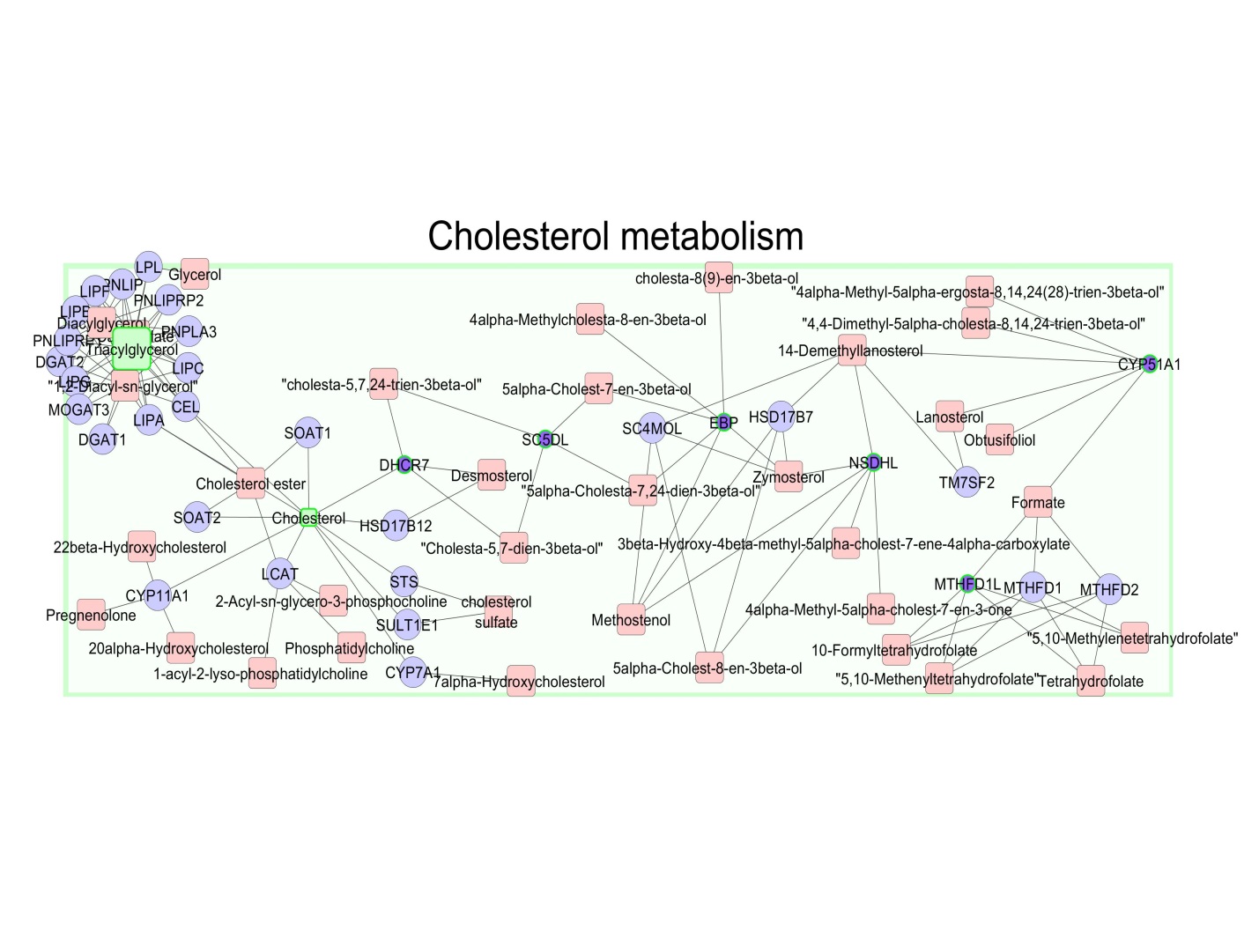
**
